# Sugar feeding protects against arboviral infection by enhancing gut immunity in the mosquito vector *Aedes aegypti*

**DOI:** 10.1371/journal.ppat.1009870

**Published:** 2021-09-02

**Authors:** Floriane Almire, Selim Terhzaz, Sandra Terry, Melanie McFarlane, Rommel J. Gestuveo, Agnieszka M. Szemiel, Margus Varjak, Alma McDonald, Alain Kohl, Emilie Pondeville

**Affiliations:** 1 MRC-University of Glasgow Centre for Virus Research, Glasgow, United Kingdom; 2 Division of Biological Sciences, University of the Philippines Visayas, Miagao, Iloilo, Philippines; Washington State University, UNITED STATES

## Abstract

As mosquito females require a blood meal to reproduce, they can act as vectors of numerous pathogens, such as arboviruses (*e*.*g*. Zika, dengue and chikungunya viruses), which constitute a substantial worldwide public health burden. In addition to blood meals, mosquito females can also take sugar meals to get carbohydrates for their energy reserves. It is now recognised that diet is a key regulator of health and disease outcome through interactions with the immune system. However, this has been mostly studied in humans and model organisms. So far, the impact of sugar feeding on mosquito immunity and in turn, how this could affect vector competence for arboviruses has not been explored. Here, we show that sugar feeding increases and maintains antiviral immunity in the digestive tract of the main arbovirus vector *Aedes aegypti*. Our data demonstrate that the gut microbiota does not mediate the sugar-induced immunity but partly inhibits it. Importantly, sugar intake prior to an arbovirus-infected blood meal further protects females against infection with arboviruses from different families. Sugar feeding blocks arbovirus initial infection and dissemination from the gut and lowers infection prevalence and intensity, thereby decreasing the transmission potential of female mosquitoes. Finally, we show that the antiviral role of sugar is mediated by sugar-induced immunity. Overall, our findings uncover a crucial role of sugar feeding in mosquito antiviral immunity which in turn decreases vector competence for arboviruses. Since *Ae*. *aegypti* almost exclusively feed on blood in some natural settings, our findings suggest that this lack of sugar intake could increase the spread of mosquito-borne arboviral diseases.

## Author summary

Mosquitoes are vectors of arboviruses, such as Zika or dengue viruses, because females require a blood meal to reproduce. In addition to blood feeding, they can also feed on sugar sources to get carbohydrates for their energy reserves. Here, we demonstrate that, in the main arbovirus vector *Aedes aegypti*, sugar feeding prior to an arbovirus-infected blood meal enhances immunity in the gut which in turn protects females against viral infection. Overall, our findings reveal that sugar feeding is a crucial determinant of mosquito vector competence for arboviruses, *i*.*e*. mosquito’s ability to get infected and transmit arboviruses. As *Ae*. *aegypti* females do not feed on sugar in some natural settings, our findings highlight that this could be in part responsible for the high susceptibility and transmission of arboviruses by this mosquito species.

## Introduction

Male and female adult mosquitoes regularly feed on plant nectar, thus intaking carbohydrates for their energy reserves [1,2]. In addition, many mosquito species, including *Aedes aegypti* (*Ae*. *aegypti*), have evolved towards anautogeny, *i*.*e*. adult females require a blood meal to develop their eggs. Indeed, ingestion of blood activates neuro-endocrine and metabolic cascades leading to the synchronous development of up to hundreds of eggs which are laid two to three days after blood feeding. The blood meal is also largely used to provide proteins (*i*.*e*. amino acids), necessary for the synthesis of yolk proteins and for reproductive output [3]. This blood meal requirement for reproduction, and the fact that a female mosquito can take several blood meals throughout its adult life, results in *Ae*. *aegypti* being a vector of numerous arthropod-borne viruses (arboviruses). When a mosquito ingests a blood meal from an arbovirus-infected vertebrate host, the arbovirus initially infects the mosquito gut, then disseminates to other tissues before finally reaching the salivary glands. Once the salivary glands are infected, the arbovirus can be transmitted to a new vertebrate host during a subsequent blood meal. Arboviruses transmitted by mosquitoes, such as dengue (DENV), chikungunya (CHIKV), and Zika (ZIKV) viruses, constitute a substantial worldwide public health threat and economic burden with an increasing population at risk due to an expansion of their geographical range and an unprecedented emergence of epidemic arboviral diseases [4–8].

The efficiency of mosquito-borne disease transmission under natural conditions is referred to as the vectorial capacity [9]. Mathematical modelling of vectorial capacity shows that the spread of mosquito-borne pathogens is highly dependent on mosquitoes [9], including their vector competence, which is the mosquito’s ability to become infected following an infectious blood meal and subsequently transmit the pathogen [10]. Therefore, unravelling the factors which make a mosquito a competent vector will ultimately increase our fundamental understanding of mosquito-borne disease emergence and spread. In addition, it will help the development of effective and sustainable vector control strategies aimed at blocking pathogen transmission. Mosquito immunity is an important factor, among others, influencing mosquito vector competence for arboviruses [11–13]. Mosquitoes possess different immune pathways that can control arbovirus infection [14–16]. This includes the NF-κB Toll [17,18] and immune deficiency (Imd) [19,20] pathways, the Janus kinase/signal transducers and activators of transcription (JAK-STAT) pathway [17,20,21], and the prophenoloxidase cascade [22]. In addition to these immune pathways, RNA interference pathways also play an important antiviral role in mosquitoes. The exogenous small interfering (exo-siRNA) pathway in particular is a major antiviral pathway limiting the replication of many arboviruses [23,24]). Finally, the PIWI-interacting RNA (piRNA) pathway, and more particularly the PIWI4 protein, is involved in the regulation of arboviral replication [25–27].

The particular mode of nutrition of mosquitoes linked to their extreme adaptation (*i*.*e*. anautogeny), alternating between sugar (carbohydrates for energy) and blood (proteins for egg development), makes them a useful animal model to fundamentally understand how intake of different nutrients affects the female physiology. While the influence of sugar and blood feeding on mosquito survival and reproduction, two other important determinants of vectorial capacity, have largely been investigated [2,3,28,29], the impact of sugar feeding on mosquito immunity and vector competence for arboviruses has not been analysed yet. Here, we investigated the effect of sugar feeding on immunity and susceptibility to viral infection in the main arbovirus vector, *Ae*. *aegypti*. We show that sugar feeding increases and maintains expression levels of genes involved in antiviral pathways in the digestive tract of females. The three major sugars naturally found in plant nectar—sucrose, glucose and fructose—can all increase antiviral gene expression. Our data show that the gut microbiota does not mediate sugar-induced immunity but partly inhibits it. Importantly, sugar intake prior to an arbovirus-infected blood meal further protects females against infection with arboviruses from different families, Semliki Forest Virus (SFV, *Alphavirus*, *Togaviridae*) and ZIKV (*Flavivirus*, *Flaviviridae*). We further show that sugar feeding blocks arbovirus initial infection and dissemination from the gut, lowers infection prevalence and intensity, thereby decreasing the transmission potential. Finally, we demonstrate that the antiviral action of sugar is mediated through the sugar-induced immunity. Overall, our findings uncover that sugar feeding is a crucial factor influencing mosquito vector competence for arboviruses, and this might affect the spread of arboviruses by *Ae*. *aegypti* mosquitoes.

## Results

### Sucrose feeding increases the expression levels of antiviral genes in the digestive tract of females

**Fig 1:**
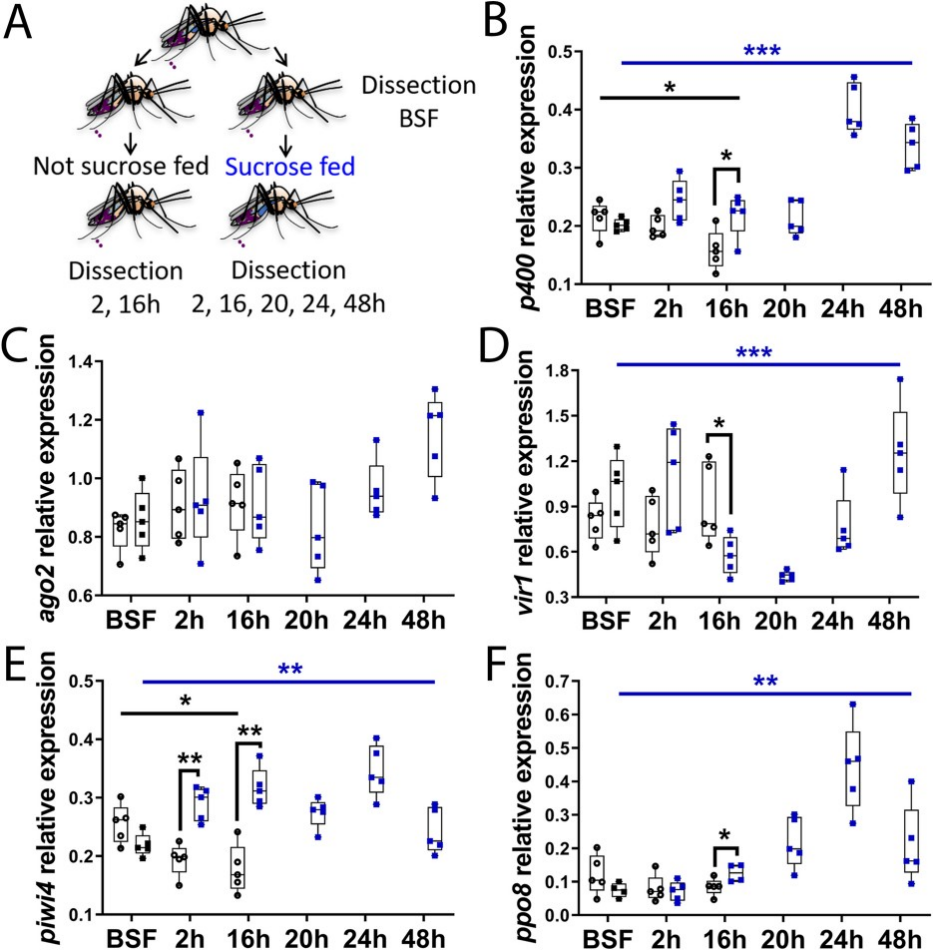
Sucrose feeding increases the expression levels of antiviral genes in the digestive tract of the female *Ae*. *aegypti*. (A) Schematic of experimental design. Females that did not have access to sucrose for 48 h were fed or not with 10% sucrose solution. Digestive tracts from both populations were dissected just before sucrose feeding (BSF) and at 2 and 16 h post sucrose feeding time, and at 20, 24 and 48 h for the sucrose fed females. (B to F) RNA transcript levels of (B) *p400*, (C) *ago2*, (D) *vir1*, (E) *piwi4* and (F) *ppo8* (black empty dots and black squares, non-sucrose fed; blue squares, sucrose fed). Box plots display the minimum, first quartile, median, third quartile, and maximum relative expression levels. Data were analysed by Mann-Whitney test (non-sucrose vs sucrose fed at each time point, BSF, 2 and 16 h) and by Kruskal-Wallis test (all times, for non-sucrose fed and sucrose fed, black and blue bars respectively showing p value summary). N = 5 pools of 5 digestive tracts per condition. Only p values < 0.05 are shown. *, p value < 0.05; **, p value < 0.01; ***, p value <0.001.

First, we investigated whether sugar could affect immunity in the female digestive tract. After a sugar meal, the ingested solution is initially stored in the mosquito crop. From there, the sugar solution is then periodically relocated from the crop to the midgut for digestion according to metabolic/physiological needs (S1 Fig; [2,30,31]). After about two days (as observed in our conditions and previously described in [2]), the crop of nearly every female is empty of all sugar solution previously ingested. Therefore, in order to compare females able to relocate and digest sugar to females that cannot, females were not given access to sucrose solution for 48 h and were then split into two groups. One group was given a 10% sucrose meal for an hour and only sugar fed (SF) females were further kept in this group. The other group was kept without sugar solution (non-sugar fed, NSF). The digestive tracts of NSF and SF females were dissected at different time points following sucrose feeding time (Fig 1A). As NSF females started to die from about 20–24 h post sucrose feeding time (*i*.*e*. 3 days after sugar removal), only SF females were dissected at 20, 24, and 48 h post sucrose feeding. Expression levels of representative mosquito genes involved in antiviral pathways were analysed by RT-qPCR (Fig 1B–1F). These genes include *p400* and *ago2*, involved in the siRNA pathway [32,33], *piwi4* involved in the piRNA pathway [25,27], *vir1*, a responder gene of the JAK-STAT pathway [21,34], and *ppo8* involved in the phenoloxidase pathway [22,35]. While the gene expression levels were identical between the two populations just before the sugar feeding (BSF, 48 h after sugar solution removed), the expression levels of *p400*, *piwi4* and *ppo8* were higher in SF females compared to NSF females at 2 h and 16 h post sucrose feeding time, with statistical significance at 16 h (Fig 1B, 1E and 1F). The expression levels of *ago2* were unchanged (Fig 1C) and the expression levels of *vir1*, although slightly higher in SF females at 2 h post sucrose meal, were lower in SF females at 16 h (Fig 1D). The expression of all analysed genes was upregulated in SF females at 24 and 48 h after sugar feeding compared to before, with *p400*, *piwi4* and *ppo8* peaking at 24 h (Fig 1B, 1E and 1F), and *ago2* and *vir1* at 48 h (Fig 1C and 1D). The expression levels of most genes in SF females were oscillating from sugar feeding to 48 h, with an alternation of increase and decrease of levels compared to the previous time point (Fig 1). This is not linked to circadian rhythm as digestive tracts sampled either before sugar feeding time or at 24 and 48 h after sugar feeding were dissected at the same time of day. Instead, this is more consistent with sugar relocation from the crop to the gut happening periodically [2,31]. Concomitantly with an increase of immune gene expression levels in SF females over time (significant for all genes except *ago2*), a decrease of immune gene expression levels (significant for *p400* and *piwi4*) occurred in NSF females from 48 h post sugar solution removal (BSF time point) to 64 h (16 h time point). This was consistent with crops being empty about two days following sugar feeding. Altogether, these results show that sucrose feeding increases and maintains for at least about two days antiviral gene expression levels in the gut of *Ae*. *aegypti* females.

### Upregulation of immunity in the gut after a sugar meal is specifically due to sugar

**Fig 2:**
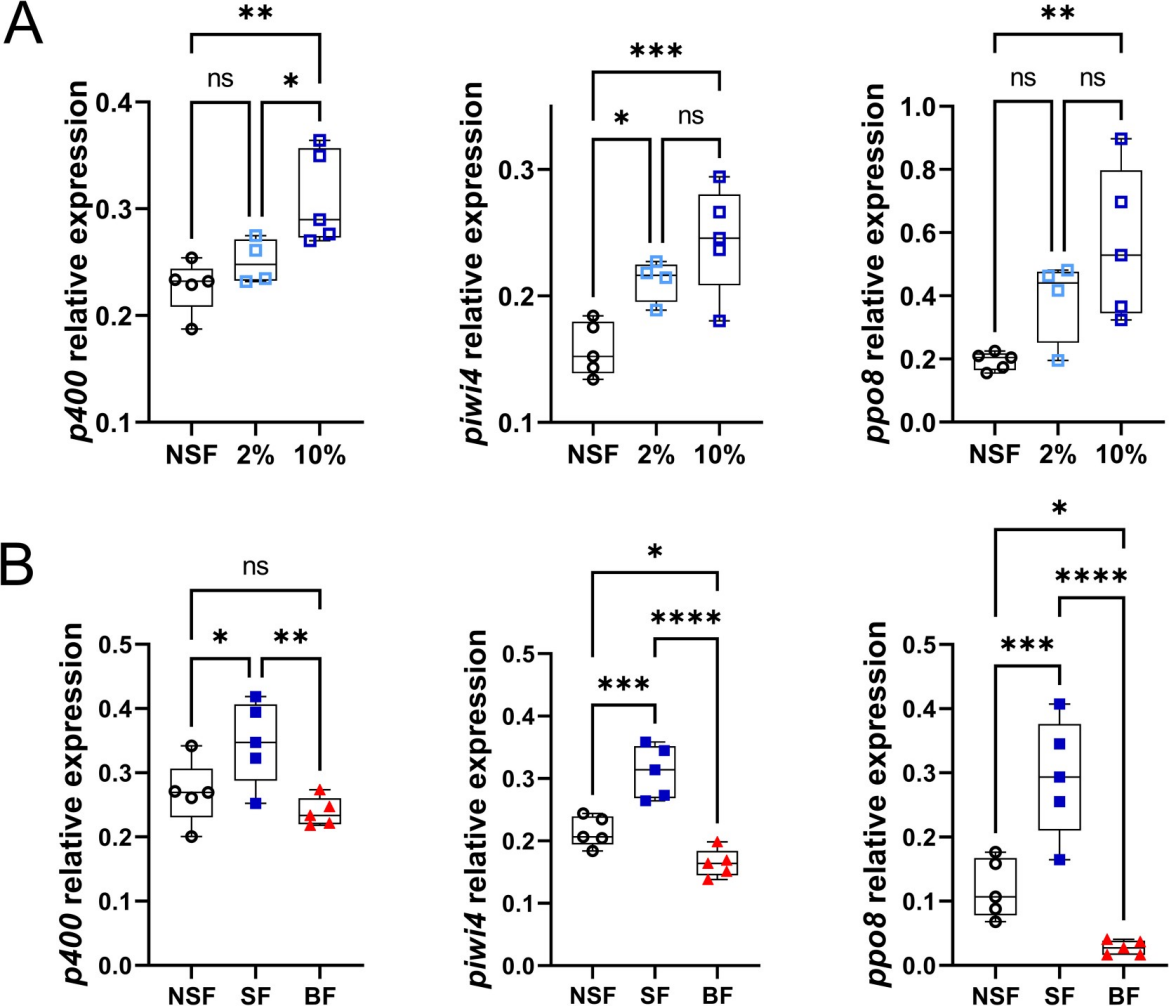
Sucrose feeding-mediated increase of antiviral genes expression level is specific to sucrose. Females were treated as in Fig 1A except that females were (A) either not fed (NSF), fed with 2% sucrose or 10% sucrose, (B) either not fed (NSF), fed with 10% sucrose (SF) or given a blood meal (BF). Digestive tracts were dissected 16 h post sucrose or blood feeding time. RNA transcript levels of *p400*, *piwi4* and *ppo8* were analysed by RT-qPCR. Box plots display the minimum, first quartile, median, third quartile, and maximum relative expression levels. Statistical significance was assessed with an analysis of variance followed by a Fisher’s multiple comparison test. N = 5 pools of 5 digestive tracts per condition. ns, p value > 0.05; *, p value < 0.05; **, p value < 0.01; ***, p value <0.001; ****, p value <0.0001.

We next wanted to assess if the difference in immune gene expression between NSF females and SF females was specifically due to sugar, to water intake or to a general effect of nutrient intake. To this aim, females were either not fed, fed with different concentrations of sucrose, or blood fed (*e*.*g*. intake of proteins). Digestive tracts were dissected at 16 h post feeding time, a time point at which the sucrose-induced antiviral gene expression was observable for *p400*, *piwi4* and *ppo8* in the previous time course experiment (Fig 1). The lower immune gene expression in NSF females compared to SF females was not due to water starvation but sucrose starvation, as the expression levels of *p400*, *piwi4* and *ppo8* in females fed with 2% sucrose were intermediary between the ones of NSF females and females fed with 10% sucrose (Fig 2A). In addition, the expression levels of *p400*, *piwi4* and *ppo8* in females were upregulated in SF females but not in blood fed females (Fig 2B). Therefore, the increase in immune gene expression in the gut of *Ae*. *aegypti* females following sugar feeding is sugar specific.

### Glucose and fructose increase the expression of antiviral genes in the digestive tract of females

**Fig 3:**
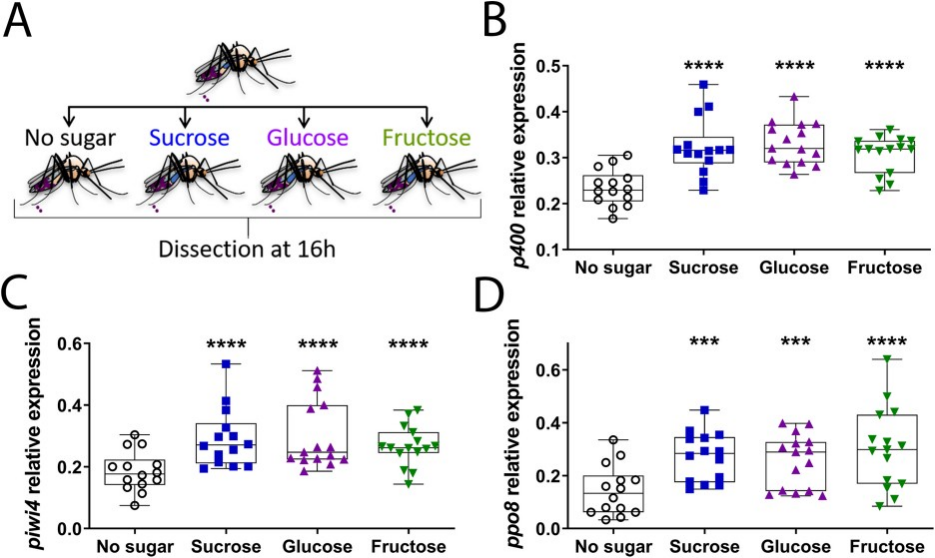
Sucrose, glucose and fructose increase the expression levels of antiviral genes in the digestive tract of the female *Ae*. *aegypti*. (A) Schematic of experimental design. Females that did not have access to sucrose for 48 h were either not sugar fed or fed with 10% sucrose, 10% glucose or 10% fructose solution. Digestive tracts were dissected 16 h post sugar feeding time. (B to D) RNA transcript levels of (B) *p400*, (C) *piwi4* and (D) *ppo8*. Box plots display the minimum, first quartile, median, third quartile, and maximum relative expression levels. Data from three separate experiments were combined after verifying the lack of a detectable experiment effect. N = 15 pools of 5 digestive tracts per condition. Statistical significance of the sugar feeding effect was assessed with an analysis of variance followed by a Fisher’s multiple comparison test (Sugar vs No sugar). ***, p value < 0.001; ****, p value <0.0001.

The disaccharide sucrose ingested by mosquitoes is hydrolysed into the monosaccharides glucose and fructose by α-glucosidases secreted in the saliva and bound to midgut epithelium membranes [30,36,37]. These monosaccharides, along with sucrose, are the most abundant sugars in nectar and are present in the natural sugar meals ingested by mosquitoes [37]. Therefore, we asked whether glucose and/or fructose were responsible for the sucrose-mediated increase of antiviral gene expression. Hence, females were not fed, or fed with either sucrose, glucose or fructose and digestive tracts were dissected at 16 h post sugar feeding (Fig 3A). As with sucrose, the expression levels of *p400*, *piwi4* and *ppo8* were significantly upregulated in the gut of the females fed with glucose or fructose, compared to NSF females (Fig 3B–3D). Thus, sucrose, glucose and fructose can increase the expression of antiviral genes in the digestive tract of *Ae*. *aegypti* females.

### Sugar-induced antiviral gene expression is not mediated by but inhibited by gut bacteria

**Fig 4:**
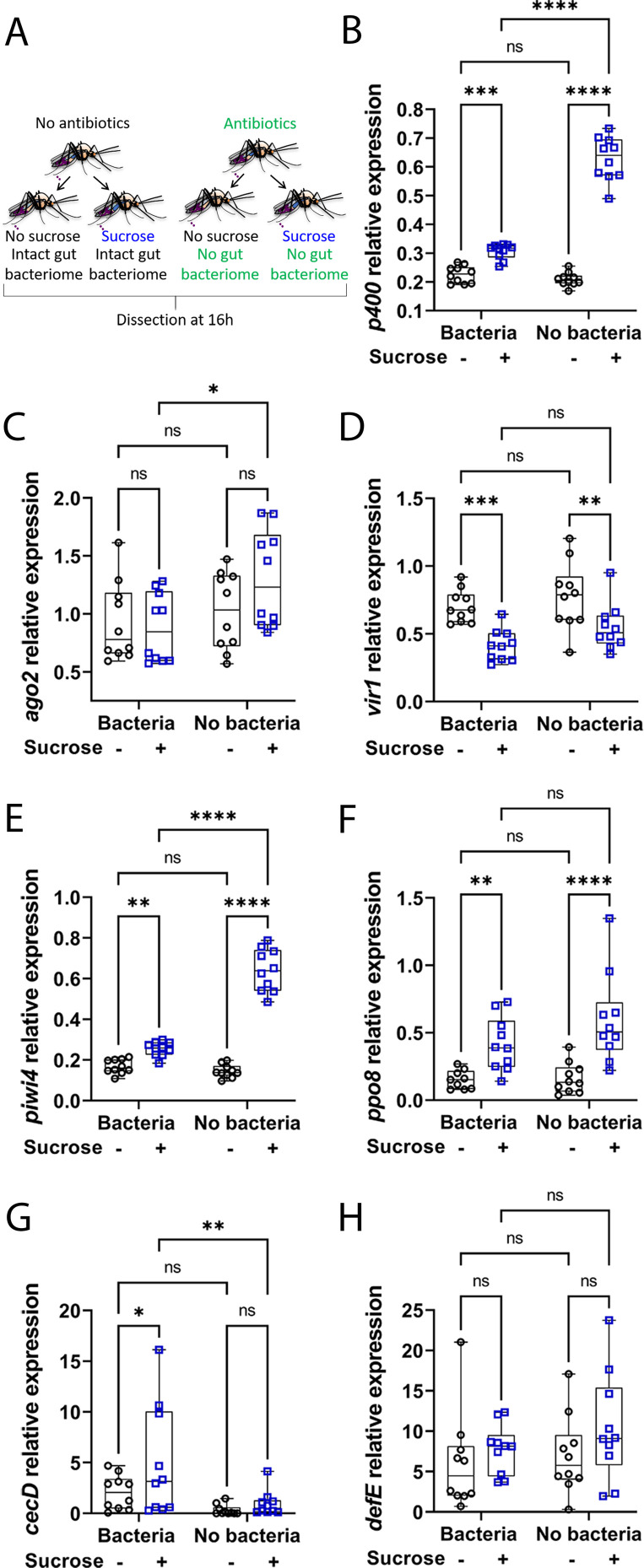
Microbiota partly inhibits sugar-induced immunity in the digestive tract of the female *Ae*. *aegypti*. (A) Schematic of experimental design. Females, previously treated or not with antibiotics, did not have access to sucrose for 48 h and were either not fed or fed with 10% sucrose. Digestive tracts were dissected 16 h post sugar feeding time. (B to H) RNA transcript levels of (B) *p400*, (C) *ago2*, (D) *vir1*, (E) *piwi4*, (F) *ppo8*, (G) *cecD* and (H) *defE*. Box plots display the minimum, first quartile, median, third quartile, and maximum relative expression levels. Data from two separate experiments were combined after verifying the lack of a detectable experiment effect. N = 10 pools of 5 digestive tracts per condition. Statistical significance of the treatments effect was assessed with a two-way ANOVA (statistical analysis summary of treatment effect and interaction between treatments given in S4 Fig). Pair-wise comparisons shown on the graphs were obtained with a post hoc Fisher’s multiple comparison test (No sucrose vs Sucrose, Bacteria vs No bacteria). ns, p value > 0.05; *, p value < 0.05; **, p value < 0.01; ***, p value <0.001; ****, p value <0.0001.

Diet, including sugar, can induce gut microbial shifts (both in terms of diversity and abundance) in different hosts [38–40]. Since endogenous gut bacteria can modulate immune gene expression in mosquitoes [41,42], we hypothesised that the increase of immune gene expression levels in the digestive tract following sugar feeding might be influenced by gut bacteria. To investigate this, the effect of sugar feeding on immune gene expression levels was compared between control mosquitoes and mosquitoes from which the gut bacteria had been eliminated through antibiotic treatment (Figs 4A and S2). As the gut microbiota can activate genes regulated by the Toll and Imd immune pathways, including antimicrobial peptides (AMPs) [18,19,43], the expression of two AMPs, *cecropin* (*cecD*) and *defensin* (*defE*), was analysed in addition to previously assessed antiviral genes at 16 h post sucrose feeding time (Fig 4B–4H). As previously observed, sucrose feeding significantly increased the expression levels of immune genes (*e*.*g*. *p400*, *piwi4* and *ppo8*) in the gut of control females (Fig 4B, 4E and 4F). Sugar feeding also significantly increased the expression of *cecD* (Fig 4G). Elimination of the gut bacteria in NSF females had little to no effect on immune gene expression except for *cecD* (Fig 4G), which was as expected expressed at lower levels in aseptic mosquitoes. Intriguingly, antibiotic treatment did not abolish the sugar-induced increase of immune gene expression but conversely led to a stronger induction of immune gene expression following sugar feeding (*p400*, *piwi4* and *ppo8*). This was specific to sugar fed females since immune gene expression was not upregulated in aseptic and blood fed females (S3 Fig). Most of the genes were expressed at higher levels in the gut of sugar fed aseptic females compared to sugar fed control females (significant for *p400*, *ago2*, *piwi4*), except *cecD* whose expression levels again decreased upon antibiotic treatment. Analyses revealed a significant overall effect of both sugar and/or antibiotic treatments for most of the genes as well as a significant interaction between sugar and antibiotic treatments for *p400* and *piwi4* (two-way ANOVA statistical significance of treatments, S4 Fig). Altogether, our results show that gut bacteria do not mediate the increase of immunity after a sugar meal, but partially inhibit the sugar-induced immune gene activation in the gut of the female *Ae*. *aegypti* mosquito.

### Sucrose intake prior to an arbovirus-infected blood meal protects females against alphavirus infection and dissemination from the gut

**Fig 5:**
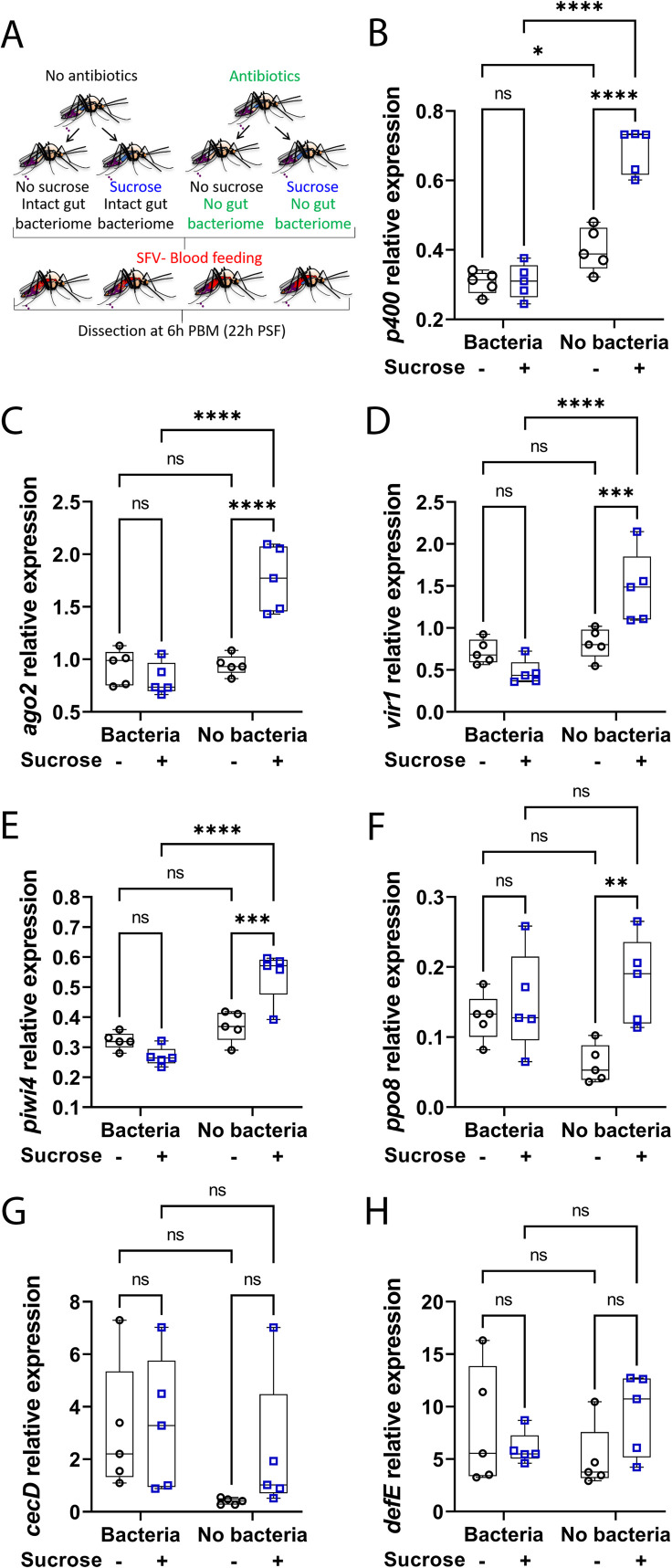
Sugar feeding prior to a blood meal increases antiviral gene expression in blood fed females. (A) Schematic of experimental design. Females, previously treated or not with antibiotics, did not have access to sucrose for 48 h and were either not fed or fed with 10% sucrose. Females received an SFV-infected blood meal at a titre of 7.8 x 10^7^ PFU/mL 16 h post sugar feeding time and digestive tracts were dissected 6 h after the blood meal. PBM, post blood meal; PSF, post sugar feeding. (B to H) RNA transcript levels of (B) *p400*, (C) *ago2*, (D) *vir1*, (E) *piwi4*, (F) *ppo8*, (G) *cecD* and (H) *defE*. Box plots display the minimum, first quartile, median, third quartile, and maximum relative expression levels. N = 5 pools of 5 digestive tracts per condition. Statistical significance of the treatments effect was assessed with a two-way ANOVA (statistical analysis summary of treatment effect and interaction between treatments given in S4 Fig). Pair-wise comparisons shown on the graphs were obtained with a post hoc Fisher’s multiple comparison test (No sucrose vs Sucrose, Bacteria vs No bacteria). ns, p value > 0.05; *, p value < 0.05; **, p value < 0.01; ***, p value <0.001; ****, p value <0.0001.

The gut is the first barrier to be overcome by an arbovirus and is considered to be a major determinant of arbovirus infection and mosquito vector competence [14,44]. After an infectious blood meal, arboviruses must rapidly infect the midgut cells before the formation of the peritrophic matrix and to avoid the proteasic environment inherent to blood digestion [44,45]. We showed that the immune gene expression in the digestive tract is enhanced following sucrose feeding and even more in the absence of the gut bacterial flora (Fig 4). Since the genes analysed here are involved in antiviral pathways in *Ae*. *aegypti*, we therefore reasoned that a sugar meal before an infectious blood meal may decrease initial arbovirus midgut infection and replication and further dissemination from the gut. To investigate this, NSF or SF females were given a blood meal infected with the arbovirus Semliki Forest virus (SFV), a prototype alphavirus of the *Togaviridae* family, 16 h after the sugar feeding time, time at which most of the immune genes analysed in this study were up-regulated following sugar feeding. As sugar-induced immunity is stronger in aseptic females, the effect of sugar feeding on infection outcome was analysed in both control and aseptic females to determine whether effects would be correlated to the strength of sugar-induced immune activation (Fig 5A). Analysis of immune gene expression in the digestive tracts at 6 h after the blood meal (Fig 5B–5H), revealed an overall significant effect of the sugar treatment on *p400*, *ago2*, *piwi4* and *ppo8* and a significant interaction between sugar and antibiotic treatment for *p400*, *ago2*, *vir1* and *piwi4* (see two-way ANOVA statistical significance of treatments in S4 Fig). Although 6 h after blood meal, sugar-mediated induction of immune gene expression was no longer detectable in the gut of females with intact bacterial flora, it was still observed in the gut of antibiotic-treated females (Fig 5B–5H). Since blood meal provokes a reduction in sugar response in the gut [2,45], it is possible that gene expression levels in SF females at the time of blood feeding (16 h post sugar feeding, Fig 4) progressively decrease after a blood meal. As the effect of sugar is stronger at the time of blood feeding (16 h post sugar feeding, Fig 4) in the gut of aseptic females compared to control ones, this may be delayed in aseptic females.

**Fig 6:**
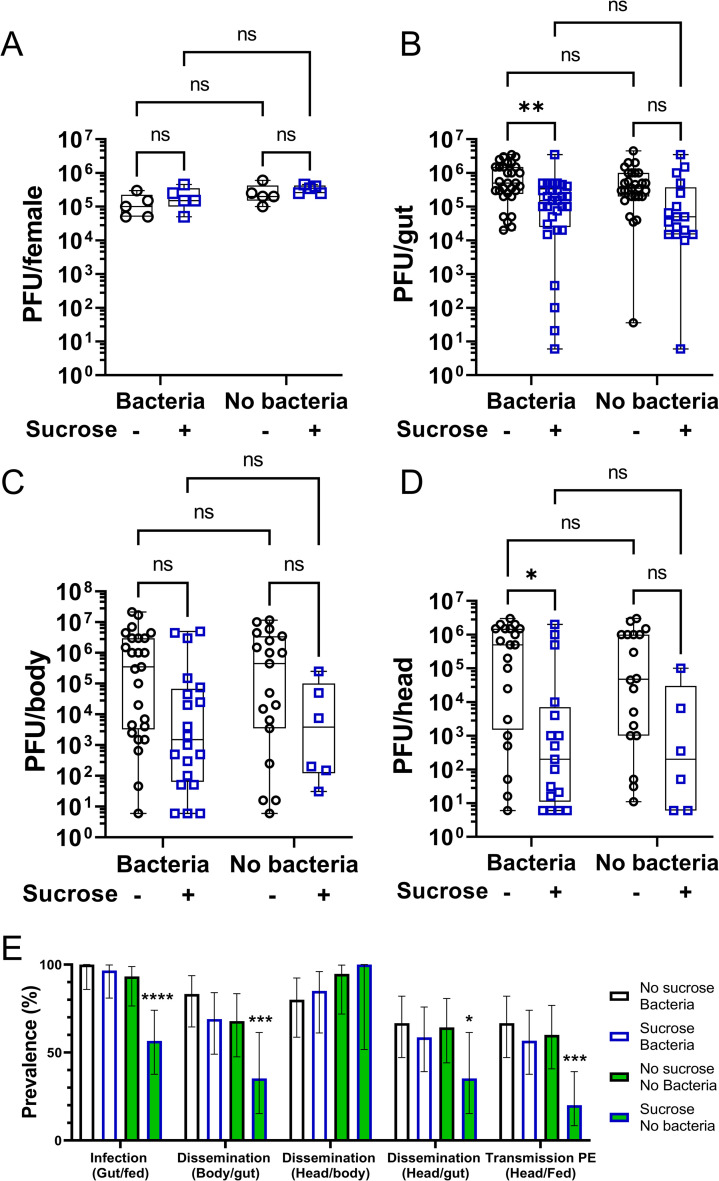
Sugar feeding protects the female mosquito *Ae*. *aegypti* against SFV infection. Females were treated as in Fig 5A. All females were given access to sucrose solution 30 h after the infectious blood meal. (A) Virus titration by plaque assays of individual females sacrificed straight after blood meal to assess infectious particles ingested. N = 5 per condition. (B to D) Virus titration by plaque assays of individual (B) guts, (C) bodies and (D) heads sampled four days post infection. N = 30 tissues per condition. Only infected samples (with 5 PFU or more per tissue, *i*.*e*. at least one plaque in one of the two replicate wells at the first dilution) were plotted on the graphs. Box plots display the minimum, first quartile, median, third quartile, and maximum. Data were analysed using two-way ANOVA (statistical analysis summary of treatment effect and interaction between treatments given in S4 Fig). Pair-wise comparisons shown on the graphs were obtained with a post hoc Fisher’s multiple comparison test (No sucrose vs Sucrose, Bacteria vs No bacteria). (E) SFV infection, dissemination and transmission potential prevalence (in percentage). Lower and upper limits of the 95% confidence interval were calculated using the Wilson score interval method with a correction for continuity. Statistical significance of the treatments effect on prevalence were assessed with a Chi-square test (compared to No bacteria—No sucrose group). ns, p value > 0.05; *, p value < 0.05; ***, p value <0.001; ****, p value <0.0001. Numbers of infected females and total number of females per group detailed in S5 Fig.

Importantly, although the quantity of initial infectious particles in females was similar between the different groups (Fig 6A), sugar feeding prior to the infectious blood meal had an overall significant effect on SFV infection (gut), dissemination (body) and transmission potential (head, as a proxy of virus in salivary glands) outcome at 4 days post infection (see two-way ANOVA statistical significance of treatments in S4 and 6B–6D Figs). In females with intact microbiota, sugar feeding significantly reduced SFV titres in infected guts and heads (Fig 6B–6D), with no effect on infection prevalence (Fig 6E). While antibiotic treatment did not influence infection titres and prevalence in NSF females, sugar feeding in aseptic females led to an even more pronounced effect on infection outcome compared to females with intact microbiota (Fig 6B–6E). Although there was only a trend for a reduction in SFV titres in infected body parts from aseptic females (Fig 6B–6D), sugar feeding strongly reduced prevalence of gut infection and dissemination to the body and head (Fig 6E). Thus, when not partially inhibited by the gut bacteria, sugar feeding increased resistance to infection, with only a small proportion of engorged females still showing a transmission potential (20%), *e*.*g*. virus dissemination to the head (Fig 6E). Infection prevalence analysis shows that once virus was in the body, sugar treatment no longer had an effect on dissemination (Fig 6E). Therefore, the impact of sugar feeding on transmission potential prevalence was only due to an effect of sugar on initial gut infection and dissemination from the gut, demonstrating that the gut is a crucial determinant in mosquito vector competence.

### Sucrose intake protects females against flavivirus infection

**Fig 7:**
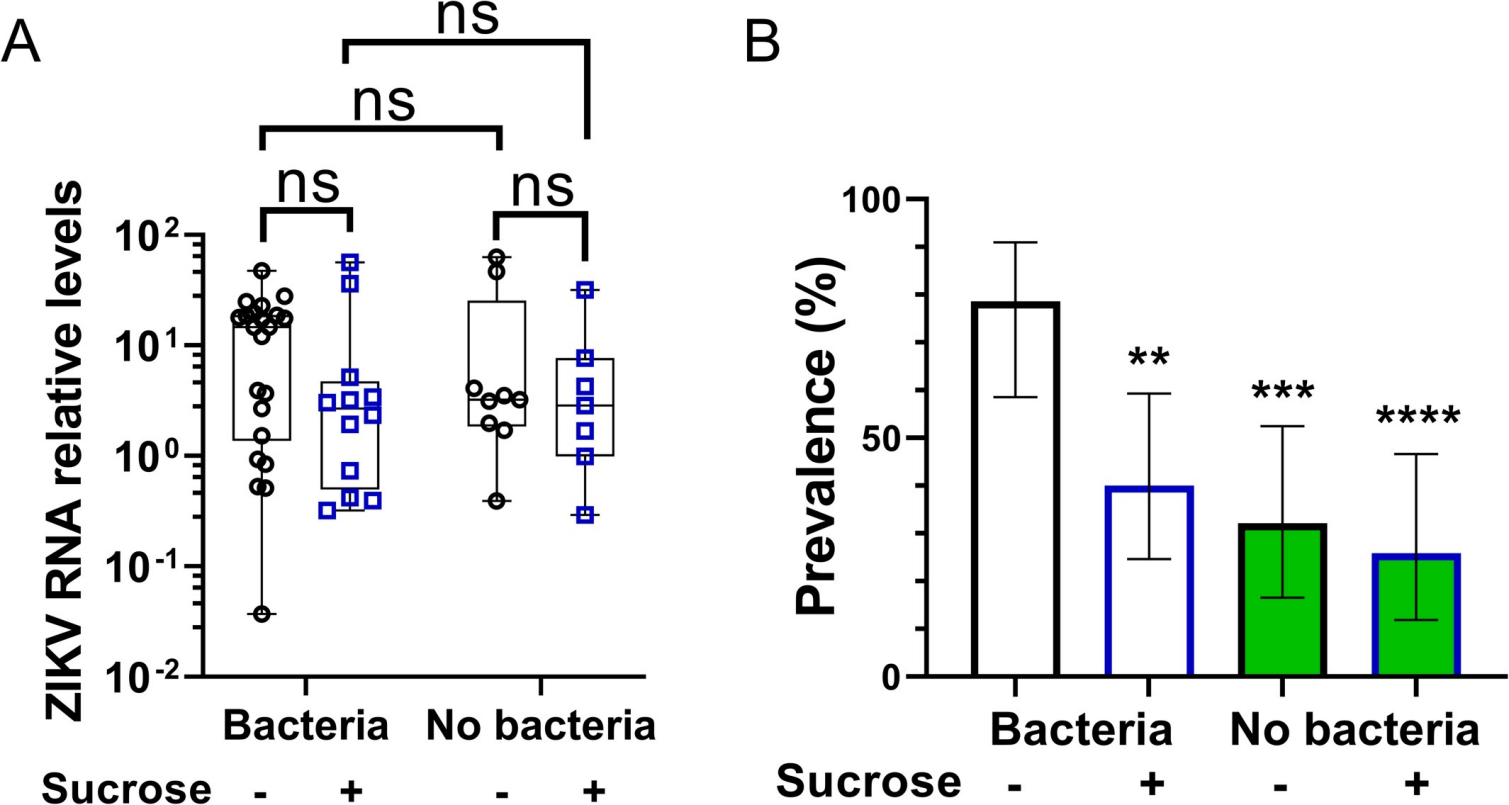
Sugar feeding protects the female mosquito *Ae*. *aegypti* against ZIKV infection. Females were treated as in Fig 5A except that females received a ZIKV-infected blood meal at a titre of 5.3 x 10^6^ PFU/mL. All females were given access to sucrose solution 30 h after infectious blood meal. (A) ZIKV RNA levels, relative to *S7 ribosomal protein* transcript, in individual whole females assessed by RT-qPCR. Data were analysed as described (88) so as the RQ geomean of the group ‘No antibiotics-No sucrose’ is 1. N = 27 to 30 females per condition. Only ‘highly infected’ samples (with Ct less than 30, samples with Ct superior to 30 were considered as weakly infected or not infected) were plotted on the graphs. Box plots display the minimum, first quartile, median, third quartile, and maximum RQ values. Log2-transformed values of RQ values were analysed using two-way ANOVA (statistical analysis summary of treatment effect and interaction between treatments given in S4 Fig). Pair-wise comparisons shown on the graphs were obtained with a post hoc Fisher’s multiple comparison test (No sucrose vs Sucrose, Bacteria vs No bacteria). (B) ZIKV infection prevalence (in percentage). Lower and upper limits of the 95% confidence interval were calculated using the Wilson score interval method with a correction for continuity. Statistical significance of the treatment effect on prevalence were assessed with a Chi-square test (compared to No bacteria- No sucrose group). ns, p value > 0.05; **, p value < 0.01; ***, p value <0.001; ****, p value <0.0001. Numbers of infected females and total number of females per group detailed in S6 Fig.

To determine if sugar feeding and its interaction with the gut bacteria could impact on infection with other arboviruses, the experiment was repeated with ZIKV (*Flavivirus*, *Flaviviridae*). Here, whole females were sampled at 6 days post infection and viral RNA levels determined by RT-qPCR (Fig 7). Sugar and antibiotic treatments as well as their interaction had little effect on ZIKV RNA levels in infected females (Figs 7A and S4). Indeed, viral RNA levels were only slightly reduced upon sugar feeding in both control and aseptic infected females (Fig 7A). Nonetheless, ZIKV infection prevalence was dramatically reduced upon sugar feeding in both control and aseptic females, and more strongly in the latter ones (Fig 7B). Interestingly, antibiotic treatment also decreased ZIKV infection prevalence in NSF females (Fig 7B), suggesting that the gut microbiota could also increase susceptibility to ZIKV infection independently of the sugar effect. Flaviviruses take longer to disseminate from the gut [46,47] than alphaviruses [48]. Since females from all groups had access to sugar 30 h after the blood meal, and that sugar-induced immunity is stronger in aseptic females, it is possible that less of these females were infected thanks to an additional protective role of sugar imbibed after the blood meal. Altogether, our findings reveal that sugar feeding before an infectious blood meal protects *Ae*. *aegypti* females against different arboviruses.

### Sugar-mediated protection against arboviral infection is mediated by sugar-enhanced immunity

**Fig 8:**
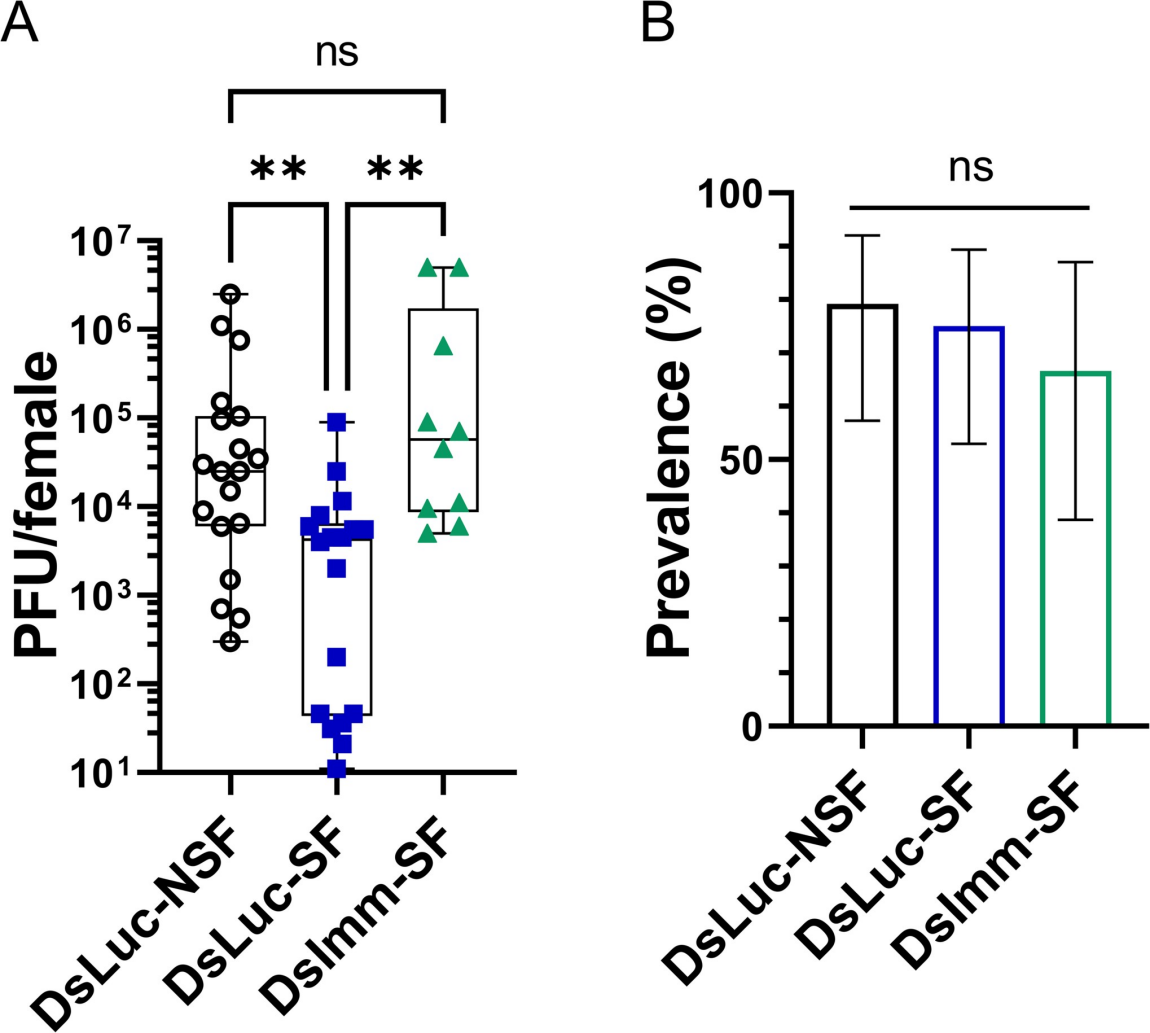
Sugar-mediated protection against arboviral infection is mediated by sugar-enhanced immunity. Females treated with antibiotics, were injected with dsRNA targeting either luciferase (dsLuc, control) or targeting *runx4*, *piwi4*, *dcr2* and *myd88* simultaneously (dsImm). Two days later, females were starved before being fed with 10% sucrose (dsLuc-SF and dsImm-SF) or not (dsLuc-NSF). Females received an SFV-infected blood meal at a titre of 7.8 x 10^7^ PFU/mL 16 h post sugar feeding time. All females were given access to sucrose solution 30 h after the infectious blood meal. (A) Virus titration by plaque assays of whole females four days post infection. N = 15–24 females per condition. Only infected samples (with 5 PFU or more per tissue, *i*.*e*. at least one plaque in one of the two replicate wells at the first dilution) were plotted on the graphs. Box plots display the minimum, first quartile, median, third quartile, and maximum. Data were analysed using Kruskal-Wallis with Dunn’s multiple comparison test. (B) SFV infection prevalence (in percentage). Lower and upper limits of the 95% confidence interval were calculated using the Wilson score interval method with a correction for continuity. Statistical significance of the treatments effect on prevalence were assessed with a Chi-square test (compared to dsLuc-NSF group). ns, p value > 0.05; **, p value < 0.01. Numbers of infected females and total number of females per group detailed in S8 Fig.

We show here that i) sugar feeding leads to inversely correlated changes between immune gene expression (increase) and virus titres/prevalence (decrease) and ii) elimination of the gut bacteria results in a higher sugar-induced immune gene activation and a stronger protection against arbovirus infection. Moreover, the immune genes analysed here are known to be antiviral or to be involved in antiviral pathways in mosquitoes including *Ae*. *aegypti* [14–16]. Altogether, it suggested that the protective effect of a sugar meal prior to an infectious blood meal on viral infection could be mediated by sugar-induced antiviral immunity. However, an immune-independent effect of sugar on virus infection could not be excluded. To determine if the sugar-induced protection against arbovirus infection was dependent on immune gene expression, we investigated whether knocking down the immune pathways could rescue virus infectivity in sugar fed females. Since sugar increases the expression of genes of different immune pathways, the most upstream or main effector protein from four different pathways were simultaneously targeted: Dicer-2 for the siRNA pathway; piwi4 for the piRNA pathway; RUNX4, a transcription factor regulating the expression of PPO genes [35]; and Myd88 for the Toll pathway [18]. While 4 days post dsRNA injection we could not detect a knockdown for *runx4*, *ppo8* expression was silenced (S7 Fig). Silencing of *dcr2*, *piwi4* and *myd88* led to a 48, 76 and 78% reduction of expression respectively (S7 Fig). The protective effect of sugar feeding against viral infection observed in SF control females (dsLuc-SF) was abolished in females whose immune pathways were knocked down (dsImm-SF) with SFV titres as high as in NSF control females (dsLuc-NSF) (Fig 8). Therefore, the protective effect of sugar against arboviral infection is mediated by the sugar-induced expression of immune genes, and the high susceptibility of NSF females is not primarily due to stress from starvation.

## Discussion

Diet profoundly influences all aspect of animal biology and physiology. In particular nutrition is a key regulator of health and disease outcomes through interactions with the immune system [49]. For instance, nutrition can impact arbovirus virulence in mice [50]. In *Drosophila*, the nutrient responsive ERK pathway is involved in the restriction of arboviral infection [51]. So far, the effect of diet on immunity and health has been mostly studied in humans and model organisms such as mice and *Drosophila melanogaster*. However, assessing the impact of diet in biomedically relevant species, such as insect disease vectors, could inform on the role of nutrition in the spread of diseases and support the design of control strategies. Here, our findings unveil that sugar feeding increases and maintains antiviral immunity in the digestive tract, and further protects the arbovirus vector *Ae*. *aegypti* against infection with different arboviruses.

It is well known that mosquito females can take sugar meals to get carbohydrates for their energy reserves, which are used for flight, survival and reproduction [2]. Here, we show that sugar meals can also increase immune gene expression in mosquito females. Consistent with our findings that in *Ae*. *aegypti* females, sugar feeding promotes the expression of immune genes, including *ppo8* which is involved in the melanisation cascade, *Anopheles* and *Culex* females are dependent on a sugar meal to mount an effective melanisation response [52,53]. A recent study has also found a positive effect of carbohydrates on immune gene expression in *Drosophila* [54]. While studies investigating the role of dietary carbohydrates on insect immunity are very scarce, some reports have highlighted a negative effect of protein and/or a positive effect of carbohydrates on resistance to infection. For instance, feeding on a high-carbohydrate diet increases survival following infection with a gut pathogen in *Drosophila* [55] and fungal infection in ants [56]. Moreover, inhibition of glucose utilisation is lethal to mice with influenza infection [57]. Although studies in more models are warranted, the protective role of sugar against infection may be conserved across evolution.

The genes analysed here are known to be antiviral or to be involved in antiviral immune pathways in mosquitoes including *Ae*. *aegypti* [14–16]. Besides, it has been previously shown that the basal levels of immune-related transcripts in the mosquito gut influence susceptibility to infection and vector competence for arboviruses [58]. Here, we show that i) sugar feeding leads to inversely correlated changes in immune gene expression (increase) and virus titres/prevalence (decrease), ii) elimination of the gut bacteria results in a higher sugar-induced immune gene activation and a stronger protection against arbovirus infection, and iii) arboviral infectivity is restored in sugar fed females whose immune pathways are knocked down. Therefore, the effect of a sugar meal prior to an infectious blood meal on viral infection is mediated by sugar-induced antiviral immunity. Since four pathways were simultaneously knocked down in our experiment (Fig 8), we cannot know the relative contribution of each pathway to the sugar-mediated protection against arboviruses. As all those pathways have been individually shown in previous studies to be antiviral in *Ae*. *aegypti*, it is nevertheless likely that they all contribute to the phenotype. Notably, the decrease of transmission potential efficiency in females previously fed with sugar (proportion of heads infected on blood fed females) was only due to a reduction in initial gut infection and further dissemination from the gut. Therefore, sugar signaling/metabolism during the early stage of infection in the gut, by limiting initial susceptibility to arboviruses, is a crucial determinant of the midgut barrier and in turn of vector competence in *Ae*. *aegypti*. Similarly, the nutrient and insulin responsive ERK pathway, by restricting arbovirus infection in the gut, is a major molecular determinant of arbovirus refractoriness in *Drosophila* [59]. It is well documented that insulin and insulin signaling can influence immunity and infection in different arthropods [60] including *Anopheles* mosquitoes [61,62]. Since immune gene expression is not induced following a blood meal (our data) while the insulin and ERK pathways can be activated in the gut of blood fed females [63,64]), this suggests that the effect of sugar is not mediated through these pathways in sugar fed females. Alternatively, the effect of sugar on immune gene expression could be due to central carbon metabolism (glycolysis, the pentose phosphate pathway, and the tricarboxylic acid cycle), which is known to be required for immune activation and function [65]. In *Drosophila*, sugar-mediated survival to intestinal infection is independent of the bacterial flora [55]. Similarly, we found that sugar-induced antiviral immunity and protection against arbovirus infection were not mediated by the gut bacteria. Sugar-mediated effects were stronger in aseptic mosquitoes, showing that the beneficial action of sugar on antiviral immunity and protection against arbovirus infection can in part be inhibited by gut bacteria. Studies have highlighted the ability of mosquito-associated bacteria to reduce [18,19,43] or, similarly to our observations, increase [20,66,67] vector competence for arboviruses. Different mechanisms, such as the modulation of immunity, have been proposed to explain the influence of bacteria on mosquito vector competence [41]. In our study, gut bacteria did not affect immunity in NSF nor in blood fed females but only in SF females. Although it cannot be excluded that bacteria inhibition is independent of the sugar activation effect, *i*.*e*. if basal levels of immunity in NSF females were too low to observe inhibition by bacteria, it suggests that microbiota could directly inhibit the sugar induction of immune gene expression in SF females. In *D*. *melanogaster*, the microbiota has been shown to shape the host nutritional requirements and to decrease sugar response and metabolism [68,69]. Different mechanisms by which bacteria modulate nutrition and metabolism have been identified such as direct competition for nutrients uptake between bacteria and the host [70] or direct manipulation of host nutrients signaling pathways by bacteria [71,72]. Interestingly, antibiotic treatment influences the tricarboxylic acid cycle in *Anopheles*, which was suggested to be linked to an increase in glycolysis [73]. Further studies will be required to identify the mechanism(s) by which sugar increases immunity and how microbiota inhibit it.

Although it is widely reported that in nature, male and female mosquitoes feed on sugar sources to maintain their energy reserves, the degree to which *Aedes* female mosquitoes require sugar meals remains unclear. Some studies have shown that *Aedes* mosquitoes seek nectar throughout their lifetime [74–76], while others have found that *Aedes* females feed rarely on sugar [77] and almost exclusively feed on blood [78]. Whether mosquito females take sugar meals or not appears to be dependent on different factors, including the availability of sugar sources [79]. Because non-sylvatic *Ae*. *aegypti* mosquitoes are adapted to human environment and often found indoors, they appear to bite more frequently and throughout a single gonotrophic cycle, acquiring multiple blood meals that are used for reproduction, energy, and survival [78,80–82]. Overall, our findings highlight that the absence of sugar feeding in some natural settings is likely increasing susceptibility and vector competence of *Ae*. *aegypti*.

Gaining a better understanding of how nutrition influences immunity and resistance to infection in biomedically relevant species is a significant challenge. Overall, our study unveils the nutritional regulations of innate immune gene expression and resistance to arbovirus infection in mosquitoes. Analysis of vector competence is often done in laboratories, where females can feed *ad libitum* on a sugar solution. It is interesting to note that it is a common practice to remove the sugar solution a few hours to a day before a blood meal (infectious or not). Therefore, our results also shed light on unsuspected factors that could influence and introduce variations in the outcome of laboratory-based infection experiments. More importantly, our findings increase our fundamental understanding of the factors influencing vector competence. Sugar feeding can also influence mosquito blood feeding frequency, survival and reproduction, which can impact on vectorial capacity [2]. However, whether sugar feeding affects the vectorial capacity and pathogen transmission rate is still unclear [1,2,28]. The role of sugar feeding in vector competence may also affect transmission rates of mosquito-borne arboviral diseases and therefore should be included in future epidemiological models. Ultimately, this could inform the development and application of vector control strategies, such as sugar baits, aimed at reducing arbovirus transmission.

## Material and methods

### Mosquito rearing

*Ae*. *aegypti* Paea strain (a gift of Dr A-B. Failloux, Institut Pasteur, France) was reared at 28°C and 80% humidity conditions with a 12 h light/dark cycle. Larvae were reared in water and fed with dry cat food (Friskies). Emerging adult mosquitoes were maintained on a 10% (w/vol) sucrose solution *ad libitum*. Females were fed with heparinised rabbit blood (Envigo, UK) for 1 h using a 37°C Hemotek system (Hemotek Ltd, Blackburn, UK).

### Sugar, antibiotic and blood feeding treatments

From emergence, adult mosquitoes were fed with unlimited access to 10% sucrose solution supplemented or not with 200 U/mL Penicillin/Streptomycin (Gibco) and 200 μg/mL Gentamicin (Invitrogen). Sucrose solution containing antibiotics was replaced every day. Sucrose solution was removed from the cages at 7 days post emergence for 48 h before females were given, or not, a 10% sucrose meal (Fisher Chemical) supplemented or not with antibiotics (same concentration as above), a 2% sucrose meal (Fisher Chemical), a 10% glucose (Sigma-Aldrich) solution or a 10% fructose (Sigma-Aldrich) solution, all containing 1 mg/ml of non-absorbable Erioglaucine disodium salt (Sigma-Aldrich), or a blood meal for 1 h using a 37°C Hemotek system (Hemotek Ltd, Blackburn, UK). Sugar fed (with blue abdomens) and blood fed females were isolated after feeding and further kept. All groups were kept without water solution. To analyse immune gene expression in BF females, females were blood fed 16 h post sucrose feeding time with heparinised rabbit blood (ENVIGO) for 1 h using a 37°C Hemotek system (Hemotek Ltd, Blackburn, UK). To analyse immune gene expression, the digestive tracts of female mosquitoes (N = 5 pools of 5 tissues per condition and per independent experiment) were dissected in PBS 1X with 0.05% Tween 20, sampled in RNA later (Sigma-Aldrich) and stored at 4°C until RNA extraction.

### Antibiotic efficacy validation

Antibiotic efficacy was controlled by both Luria-Bertani (LB) plating 16 h post sucrose feeding time and 16S qPCR 18 h post sucrose feeding time. Prior to dissection of the digestive tracts used to control the efficacy of antibiotics, females were washed for 10 sec with three consecutive baths of 70% ethanol and one bath of PBS 1X. For LB plating, five digestive tracts were pooled (per condition and per independent experiment) in 200 μL of PBS 1X and homogenized (Precellys 24, Bertin Technologies) at 6500g for 30 sec. Colony forming units were determined by plating the homogenate of the digestive tracts with series dilution on LB-agar medium under aseptic conditions and incubated at 37°C for 24 h. For 16S qPCR, five digestive tracts (per condition and per independent experiment) were pooled in 180 μL PBS 1X and were homogenized at 6500g for 30 sec. Extraction of gDNA was performed using the DNeasy blood and tissue kit (Qiagen) following the manufacturer protocol. gDNA concentration as well as optic densities (OD) 260/280 and 260/230 was measured using a nanodrop and gDNA was aliquoted and stored at -20°C until qPCR.

### dsRNA production and injection

Total RNA was extracted with TRIzol (Thermo Fisher Scientific) from whole *Ae*. *aegypti* females according to manufacturer’s instructions including DNase treatment (TURBO DNase, Ambion). cDNA was generated from 1 μg of total RNA using MMLV (Thermo Fisher Scientific) and random hexamers (Thermo Fisher Scientific). cDNAs were further amplified with KOD Hot Start Master Mix (EMD Millipore). Gene-specific primers (S1 Table) with T7 RNA polymerase promoter sequence were used to amplify a gene-derived fragment, and further purified using the QIAquick Gel Extraction kit (Qiagen). After sequencing, the PCR product was used as a template for a second PCR using the same primers and polymerase. For production of dsLuciferase (dsLuc, used as control dsRNA), specific primers with T7 RNA polymerase promoter sequences were used to amplify a Luc-derived fragment from pGL3 Luciferase plasmid (Promega) containing the luciferase gene. dsRNAs were synthesised and purified using the MEGAscript RNAi kit (Thermo Fisher Scientific) according to the manufacturer’s instructions. dsRNA was then purified and concentrated to 10 μg/μl in nuclease free water using Sodium Acetate (3M) (Ambion) and ethanol precipitation. At 1 to 2 days after emergence, cold-anesthetised female mosquitoes (previously treated with antibiotics) were injected with dsRNA into their thorax using a nanoinjector (Nanoject II, Drummond Scientific) with 2 μg of dsRNA (dsLuc or simultaneous injection of dsDcr2, dsRunx4, dsPiwi4 and dsMyd88, 500 ng each) in 414 nl of injection solution. To maximise knockdown efficiencies, Cellfectin II transfection reagent (Thermo Fisher Scientific) was added to the injection solution as described in [83]. Females were let to recover with unlimited access to 10% sucrose solution supplemented with antibiotics for two days before being starved for downstream experiments.

### Cell culture

BHK-21 cells (a kind gift of Prof. R. M. Elliott, MRC-University of Glasgow Centre for Virus Research, UK) were cultured in GMEM (Gibco), 10% FBS, 10% TPB (Gibco) and 83 U/ml penicillin/streptomycin (Gibco). A549 NPro cells (a kind gift of Prof. R. E. Randall, University of St. Andrews, UK) were maintained in DMEM (Gibco) with 10% FBS, and expression of bovine viral diarrhea virus-derived NPro, which targets IRF-3 for degradation [84,85] was maintained by the addition of 2 μg/ml puromycin (Gibco). Vero E6 cells (ATCC#CRL-1586; a kind gift of Prof. M. Bouloy, Institut Pasteur, France) were maintained in DMEM (Gibco) supplemented with 10% FBS. All cells were maintained at 37°C in a humidified incubator with 5% CO_2_.

### Virus production

SFV4 was rescued from plasmid pCMV-SFV4 as described previously [86]. Briefly, the plasmid was transfected using Lipofectamine 2000 (Thermo Fisher Scientific) into BHK-21 cells grown in GMEM with 2% FBS and 10% TPB at 37°C with 5% CO_2_. Harvested virus stock was amplified in BHK-21 cells in GMEM supplemented with 2% FBS and 10% TPB for 2 days; at harvest, the cellular debris was spun down by centrifugation at 4000 rpm for 10 min, the obtained virus stock was topped up with FBS to 10% and sodium bicarbonate added (1 ml of 7.5% solution per 20 ml of stock). The virus stock was aliquoted and stored at -80°C and used in subsequent experiments. ZIKV virus, rescued from pCCL-SP6-ZIKVwt [87], was obtained from Jamie Royle (University of Glasgow Centre for Virus Research). The stock was amplified in A549 NPro cells for 8 days in DMEM supplemented with 2% FBS, and 25 mM HEPES. Upon harvest, the supernatant was clarified by centrifugation at 4000 rpm for 10 min. The supernatant was then concentrated on Amicon Ultra 15 filter columns (Merck Millipore). The obtained virus stock was topped up with FBS to 10% and sodium bicarbonate added. The virus stock was aliquoted and stored at -80°C.

### SFV and ZIKV infections

Females treated with sucrose supplemented (or not) with antibiotics since emergence were not given access to sucrose solution for about 48 h before receiving (or not) a 10% sucrose solution supplemented (or not) with antibiotics. An SFV- or ZIKV-infected blood meal was offered to females at 16 h post sucrose feeding time. To prepare the infectious blood meal, fresh rabbit blood (Envigo) was washed with PBS to remove white blood cells and serum. PBS was then added to the red blood cell fraction to return to the initial blood volume. The infectious blood meal was prepared with 2/3 of washed blood and 1/3 of virus-containing culture medium to give a final titre of 7.8 x 10^7^ PFU/mL for SFV or 5.3 x 10^6^ PFU/mL for ZIKV, supplemented with 2 mM ATP. Only engorged females were further kept at 28°C and 80% humidity. Access to 10% sucrose solution *ad libitum* was permitted from 30 h post infectious blood meal. SFV-infected females (N = 5 individual females per condition and per independent experiment) were sampled just after the infectious blood meal, homogenized in 100 μL of GMEM (Gibco) containing 10% FBS and stored at -80°C until titration to assess ingested virus quantity. To analyse immune gene expression, the digestive tracts of SFV-infected female mosquitoes (N = 5 pools of 5 digestive tracts per condition and per independent experiment) were dissected in PBS with 0.05% Tween 20, sampled in RNA later (Sigma-Aldrich) and stored at 4°C until RNA extraction. SFV-infected females were sampled four days post infection (N = 30 per condition and per independent experiment). Heads, digestive tracts and the remains of bodies were individually homogenized in 100 μL of GMEM (Invitrogen) containing 10% FBS and stored at -80°C until titration. Whole ZIKV-infected females were sampled six days post infection, homogenized individually (N = 30 per condition and per independent experiment) in 500 μl of TRIzol (Invitrogen) and stored at -80°C until RNA extraction.

### Virus titration

SFV4 stock was titrated by plaque assay on BHK-21 cells. For titration, 10-fold serial dilutions of virus stock were prepared in GMEM containing 2% FBS. Following an hour-long incubation with inoculum, the cells were overlaid with 1X MEM (Gibco) containing 2% FBS and 0.6% Avicel (FMC Biopolymer). The cells were incubated for 2 days at 37°C with 5% CO_2_, and thereafter fixed using equal volume of 8% formaldehyde solution for 1 h. Plaques were stained using 0.2% toluidine blue (Sigma-Aldrich). The titre of infectious particles is expressed as plaque forming units per ml (PFU/mL). ZIKV stock was titrated by plaque assay on Vero E6 cells. For titration, 10-fold serial dilutions of virus stock were prepared in PBS containing 2% FBS. Following an hour-long incubation with inoculum, cells were overlaid with 1X MEM (Gibco) containing 2% FBS and 0.6% Avicel. Cells were incubated for 5 days at 37°C with 5% CO_2_, and thereafter fixed using equal volume of 8% formaldehyde solution for 1 h. Plaques were stained using crystal violet solution (20% (v/v) ethanol, 1% (v/v) methanol, 0.1% (w/v) methyl violet (Fisher Scientific), diluted in H2O). Titre of infectious particles is expressed as PFU/mL. Mosquito samples infected with SFV were serially diluted in GMEM containing 2% FBS and 1X antibiotic-antimycotic solution (Final concentration: 100 U/mL penicillin, 0.1 g/mL streptomycin, 250 ng/mL amphotericin B, Sigma-Aldrich) and inoculated onto BHK-21 cells seeded the day before at 1.5 x 10^5^ cells/ml on 24-well plates (two replicate wells per sample). Plates were incubated for 1 h at 37°C with 5% CO_2_ and were then overlaid with 1X MEM (Gibco) containing 2% FBS and 0.6% Avicel for 2 days. Plates were fixed with an equal volume of 10% Formalin (Sigma-Aldrich) and plaques were revealed using 0.2% toluidine blue (Sigma-Aldrich). The average number of plaques (from 2 wells) was multiplied by the dilution factor to obtain the titre of infectious particles as PFU/tissue. Samples were considered negative when no plaque was obtained in the two replicate wells (*i*.*e* less than 5 PFU/sample).

### RNA extraction and reverse-transcription

RNA later was removed from the samples. Samples were homogenized (Precellys 24, Bertin Technologies) in 1 mL of TRIzol (Invitrogen), or in 600 μL of buffer RLT (Qiagen) for digestive tracts containing blood, at 6500g for 30 sec. Total RNA was extracted using the TRIzol method (Invitrogen) according to the manufacturer’s protocol except that 1-Bromo-3-ChloroPropane (BCP) (Sigma-Aldrich) 1 M was used instead of chloroform. DNase treatment was performed during 30 min at 37°C following the manufacturer’s protocol (TURBO DNase, kit Invitrogen), except that RNasine 0.36 U/μL (Promega) was also added. For digestive tracts containing blood, total RNA was extracted with the QIAamp RNAeasymini kit (Qiagen) following the manufacturer’s protocol. The recommended DNase I treatment (Qiagen) was also performed. RNA concentration was then measured using a nanodrop. Complementary DNAs (cDNAs) were synthesized from 25 ng/μL of total RNA using M-MLV Reverse Transcriptase (Invitrogen) or water (for negative RT) following the manufacturer’s protocol. Standard cDNAs were produced from 25 ng/μL of total RNA from whole females and were then diluted at 1:1000, 1:100, 1:10 and 1:1. All cDNAs were aliquoted and stored at -20°C until qPCR.

### qPCR

qPCR assays were performed with the Fast SYBR Green Master Mix method (Thermo Fisher Scientific) according to the manufacturer’s protocol and using specific primers (Sigma-Aldrich) for genes of interest (S1 Table). Real-time qPCRs were run on an ABI 7500 Fast RT PCR machine and results were analysed with the 7500 Software v2.0.6. To quantify immune gene expression, data were analysed using the relative standard curve method. The average value of technical triplicates was normalized to the *S7* ribosomal average value for each sample and each gene. All qPCR within one experiment and independent replicates were performed using the same standard cDNAs batch. To quantify ZIKV RNA levels, qPCR assays were run with the comparative Ct (cycle threshold) method using *S7 ribosomal protein* gene as a standard gene for normalisation and according to the Taylor method [88] to obtain a geomean of RQ = 1 for the control group (no antibiotics-no sucrose) and relative RQ values for every other sample. To measure *16S* gene levels, 10 ng/μL of gDNA were used. qPCR assays were run with the comparative Ct method using *S7 ribosomal protein* gene as a standard gene for normalisation and according to the Taylor method [88] to get a geomean of RQ = 1 for the control groups (no antibiotics) and relative RQ values for every other sample.

### Statistics

Statistic results were obtained using a statistical software package (GraphPad Prism 9). For time course experiments, a Mann-Whitney test was performed for time point comparisons and a Kruskal Wallis test to test for the effect of time per treatment. Models including interactions (experiment X sugar X bacteria) were analysed with type-III ANOVA, whereas models without interactions (experiment X sugar or sugar X bacteria) were analysed with type-II ANOVA. Interactions with the experiment term were removed from the model as they were not statistically significant (p > 0.05). The p values indicate statistical significance of the treatment assessed with an ANOVA accounting for the experiment effect if there was one. Post hoc Fisher’s multiple comparisons were performed to get statistical significance of the desired pair-wise comparisons. For knockdown efficiency, the p values indicate statistical significance of the treatment assessed with an unpaired t test two-tailed. For the viral infectivity rescue, data were analysed using Kruskal-Wallis with Dunn’s multiple comparison test. For relative mRNA or gene detection using the comparative Ct method, Log2-transformed values of RQ values were used for statistical analyses. For prevalence of infection, lower and upper limits of the 95% confidence interval were calculated using the Wilson score interval method with a correction for continuity. Statistical analyses of the infection prevalence were performed using a Chi-square test using the proportion of infected samples and the number of total samples per analysed group.

## Supporting information

S1 Fig Sucrose solution is stored in the crop and intermittently relocated to the midgut. Pictures of digestive tracts dissected from females fed with a blue-stained 10% sucrose solution. (A) Picture of a representative gut minutes after sugar feeding showing sugar stored in the crop and relocated to midgut. (B) Picture of a representative gut one day after sugar feeding, showing sugar in the crop (although less than just after feeding) and little to no sugar solution in the midgut.
(DOCX)

S2 Fig Validation of antibiotic treatment efficacy. Females, previously treated or not with antibiotics, did not have access to sucrose for 48 h to empty their crops and were then either not fed or fed with 10% sucrose. Digestive tracts were dissected (A to D) 16 h or (E) 18 h post sugar feeding time (E). (A to D) LB agar plates after plating homogenised and diluted (1/100) digestive tracts from (A) non fed females and (B) sucrose fed females not treated with antibiotics and digestive tracts from (C) non-fed females and (D) sucrose fed females treated with antibiotics. (E) *16S* relative gene levels in digestive tracts from the four populations, relative to *S7 ribosomal protein* gene, were analysed by qPCR. RQ for each sample was obtained as described (88), normalised to the *S7* ribosomal gene and as relative values to that of the respective control group (not treated with antibiotics, RQ geomean set to 1). Box plots display the RQ minimum, first quartile, median, third quartile, and maximum. Log2-transformed RQ values were analysed by ANOVA on matched values followed by a Holm-Sidak’s multiple comparison test. Bacteria in the digestive tracts of antibiotic-treated females were significantly depleted. Samples were similarly depleted of bacteria (No sucrose vs Sucrose: ns). N = 3 pools (from 3 independent experiments) of 5 digestive tracts per condition.
(DOCX)

S3 Fig Immunity is not upregulated in aseptic blood fed females. Females previously treated with antibiotics did not have access to sucrose for 48 h and were either not fed (NSF), fed with 10% sucrose (SF), or blood fed (BF). Digestive tracts were dissected 16 h post feeding time. RNA transcript levels of *p400*, *piwi4* and *ppo8*. Box plots display the minimum, first quartile, median, third quartile, and maximum relative expression levels. N = 5 pools of 5 digestive tracts per condition. Statistical significance was assessed with an analysis of variance followed by a Fisher’s multiple comparison test. ns, p value > 0.05; *, p value < 0.05; ****, p value <0.0001.
(DOCX)

S4 Fig Two-way ANOVA statistical significance of treatments and interaction between treatments. Treatments: Sugar and Antibiotic (Bacteria) treatments. Significant treatment effects and interaction between treatments (p values < 0.05) are highlighted in blue.
(DOCX)

S5 Fig Sugar feeding protects the female mosquito *Ae*. *aegypti* against SFV infection. SFV infection, dissemination and transmission potential prevalence (in percentage and numbers in brackets). The p values indicate statistical significance of the treatments effect on prevalence assessed with a Chi-square test (compared to No bacteria—No sucrose group). ns, p value > 0.05. Data corresponding to graph presented on Fig 6E.
(DOCX)

S6 Fig Sugar feeding protects the female mosquito *Ae*. *aegypti* against ZIKV infection. ZIKV infection prevalence (in percentage and numbers in brackets). The p values indicate statistical significance of the treatment effect on prevalence assessed with a Chi-square test (compared to No bacteria- No sucrose group). Data corresponding to graph presented on Fig 7B.
(DOCX)

S7 Fig Knock down efficiency of *ppo8*, *piwi4*, *dcr2* and *myd88*. Females treated with antibiotics, were injected with dsRNA targeting either luciferase (dsLuc, control) or targeting *runx4*, *piwi4*, *dcr2* and *myd88* (dsImm). Two days later, females were starved before being fed with 10% sucrose. Digestive tracts were dissected 16 h post sugar feeding time. RNA transcript levels of *runx4*, *ppo8*, *piwi4*, *dcr2* and *myd88*. Box plots display the minimum, first quartile, median, third quartile, and maximum relative expression levels. N = 5 pools of 5 digestive tracts per condition. Statistical significance of the treatment effect was assessed with an unpaired t test two-tailed. ns, p value > 0.05; **, p value < 0.01; ****, p value <0.0001.
(DOCX)

S8 Fig Sugar-mediated protection against arboviral infection is mediated by sugar-enhanced immunity. SFV infection prevalence (in percentage and numbers in brackets). The p values indicate statistical significance of the treatment effect on prevalence assessed with a Chi-square test (compared to DsLuc-NSF group). Data corresponding to graph presented on Fig 8B.
(DOCX)

S1 Table List of primers used in this study. (DOCX)

## References

[1] Barredo E, DeGennaro M. Not Just from Blood: Mosquito Nutrient Acquisition from Nectar Sources. Trends Parasitol. 2020;36(5):473–84. doi: 10.1016/j.pt.2020.02.003 32298634

[2] Foster WA. Mosquito sugar feeding and reproductive energetics. Annu Rev Entomol. 1995;40:443–74. doi: 10.1146/annurev.en.40.010195.002303 7810991

[3] Clements AN. The biology of mosquitoes. Development, nutrition and reproduction.: Chapman & Hall; 1992.

[4] Weaver SC, Charlier C, Vasilakis N, Lecuit M. Zika, Chikungunya, and Other Emerging Vector-Borne Viral Diseases. Annu Rev Med. 2018;69:395–408. doi: 10.1146/annurev-med-050715-105122 28846489 PMC6343128

[5] Wilder-Smith A, Gubler DJ, Weaver SC, Monath TP, Heymann DL, Scott TW. Epidemic arboviral diseases: priorities for research and public health. Lancet Infect Dis. 2017;17(3):e101–e6. doi: 10.1016/S1473-3099(16)30518-7 28011234

[6] Shepard DS, Undurraga EA, Halasa YA, Stanaway JD. The global economic burden of dengue: a systematic analysis. Lancet Infect Dis. 2016;16(8):935–41. doi: 10.1016/S1473-3099(16)00146-8 27091092

[7] Gould E, Pettersson J, Higgs S, Charrel R, de Lamballerie X. Emerging arboviruses: Why today? One health. 2017;4:1–13. doi: 10.1016/j.onehlt.2017.06.001 28785601 PMC5501887

[8] Messina JP, Brady OJ, Golding N, Kraemer MUG, Wint GRW, Ray SE, et al. The current and future global distribution and population at risk of dengue. Nature microbiology. 2019;4(9):1508–15. doi: 10.1038/s41564-019-0476-8 31182801 PMC6784886

[9] Smith DL, Battle KE, Hay SI, Barker CM, Scott TW, McKenzie FE. Ross, macdonald, and a theory for the dynamics and control of mosquito-transmitted pathogens. PLoS Pathog. 2012;8(4):e1002588. doi: 10.1371/journal.ppat.1002588 22496640 PMC3320609

[10] Hardy JL, Houk EJ, Kramer LD, Reeves WC. Intrinsic factors affecting vector competence of mosquitoes for arboviruses. Ann Rev Entomol. 1983;28:229–62. doi: 10.1146/annurev.en.28.010183.001305 6131642

[11] Bartholomay LC, Michel K. Mosquito Immunobiology: The Intersection of Vector Health and Vector Competence. Annu Rev Entomol. 2018;63:145–67. doi: 10.1146/annurev-ento-010715-023530 29324042

[12] Ruckert C, Ebel GD. How Do Virus-Mosquito Interactions Lead to Viral Emergence? Trends Parasitol. 2018;34(4):310–21. doi: 10.1016/j.pt.2017.12.004 29305089 PMC5879000

[13] Tabachnick WJ. Nature, nurture and evolution of intra-species variation in mosquito arbovirus transmission competence. International journal of environmental research and public health. 2013;10(1):249–77. doi: 10.3390/ijerph10010249 23343982 PMC3564141

[14] Cheng G, Liu Y, Wang P, Xiao X. Mosquito Defense Strategies against Viral Infection. Trends Parasitol. 2016;32(3):177–86. doi: 10.1016/j.pt.2015.09.009 26626596 PMC4767563

[15] Wu P, Yu X, Wang P, Cheng G. Arbovirus lifecycle in mosquito: acquisition, propagation and transmission. Expert Rev Mol Med. 2019;21:e1. doi: 10.1017/erm.2018.6 30862324

[16] Sim S, Jupatanakul N, Dimopoulos G. Mosquito Immunity against Arboviruses. Viruses. 2014;6(11):4479–504. doi: 10.3390/v6114479 25415198 PMC4246235

[17] Anglero-Rodriguez YI, MacLeod HJ, Kang S, Carlson JS, Jupatanakul N, Dimopoulos G. Aedes aegypti Molecular Responses to Zika Virus: Modulation of Infection by the Toll and Jak/Stat Immune Pathways and Virus Host Factors. Front Microbiol. 2017;8:2050. doi: 10.3389/fmicb.2017.02050 29109710 PMC5660061

[18] Xi Z, Ramirez JL, Dimopoulos G. The Aedes aegypti toll pathway controls dengue virus infection. PLoS Pathog. 2008;4(7):e1000098. doi: 10.1371/journal.ppat.1000098 18604274 PMC2435278

[19] Barletta AB, Nascimento-Silva MC, Talyuli OA, Oliveira JH, Pereira LO, Oliveira PL, et al. Microbiota activates IMD pathway and limits Sindbis infection in Aedes aegypti. Parasites & vectors. 2017;10(1):103. doi: 10.1186/s13071-017-2040-9 28231846 PMC5324288

[20] Carissimo G, Pondeville E, McFarlane M, Dietrich I, Mitri C, Bischoff E, et al. Antiviral immunity of Anopheles gambiae is highly compartmentalized, with distinct roles for RNA interference and gut microbiota. Proc Natl Acad Sci U S A. 2015;112(2):E176–85. doi: 10.1073/pnas.1412984112 25548172 PMC4299212

[21] Souza-Neto JA, Sim S, Dimopoulos G. An evolutionary conserved function of the JAK-STAT pathway in anti-dengue defense. Proc Natl Acad Sci U S A. 2009;106(42):17841–6. doi: 10.1073/pnas.0905006106 19805194 PMC2764916

[22] Rodriguez-Andres J, Rani S, Varjak M, Chase-Topping ME, Beck MH, Ferguson MC, et al. Phenoloxidase activity acts as a mosquito innate immune response against infection with Semliki Forest virus. PLoS Pathog. 2012;8(11):e1002977. doi: 10.1371/journal.ppat.1002977 23144608 PMC3493465

[23] Olson KE, Blair CD. Arbovirus-mosquito interactions: RNAi pathway. Curr Opin Virol. 2015;15:119–26. doi: 10.1016/j.coviro.2015.10.001 26629932 PMC4765169

[24] Samuel GH, Adelman ZN, Myles KM. Antiviral Immunity and Virus-Mediated Antagonism in Disease Vector Mosquitoes. Trends Microbiol. 2018:447–61. doi: 10.1016/j.tim.2017.12.005 29395729 PMC5910197

[25] Varjak M, Maringer K, Watson M, Sreenu VB, Fredericks AC, Pondeville E, et al. Aedes aegypti Piwi4 Is a Noncanonical PIWI Protein Involved in Antiviral Responses. mSphere. 2017;2(3). doi: 10.1128/mSphere.00144-17 28497119 PMC5415634

[26] Schnettler E, Donald CL, Human S, Watson M, Siu RW, McFarlane M, et al. Knockdown of piRNA pathway proteins results in enhanced Semliki Forest virus production in mosquito cells. The Journal of general virology. 2013;94(Pt 7):1680–9. doi: 10.1099/vir.0.053850-0 23559478 PMC3709635

[27] Tassetto M, Kunitomi M, Whitfield ZJ, Dolan PT, Sanchez-Vargas I, Garcia-Knight M, et al. Control of RNA viruses in mosquito cells through the acquisition of vDNA and endogenous viral elements. eLife. 2019;8. doi: 10.7554/eLife.41244 31621580 PMC6797480

[28] Stone CM, Foster WA. Plant-sugar feeding and vectorial capacity. Ecology of parasite-vector interactions Ecology and control of vector-borne diseases. vol 3. Wageningen: Wageningen Academic Publishers; 2013.

[29] Clements AN. The biology of mosquitoes. Sensory reception and behaviour: CABI; 1999.

[30] Souza-Neto JA, Machado FP, Lima JB, Valle D, Ribolla PE. Sugar digestion in mosquitoes: identification and characterization of three midgut alpha-glucosidases of the neo-tropical malaria vector Anopheles aquasalis (Diptera: Culicidae). Comp Biochem Physiol A Mol Integr Physiol. 2007;147(4):993–1000. doi: 10.1016/j.cbpa.2007.03.008 17449310

[31] Stoffolano JG Jr., Haselton AT. The adult Dipteran crop: a unique and overlooked organ. Annu Rev Entomol. 2013;58:205–25. doi: 10.1146/annurev-ento-120811-153653 23317042

[32] McFarlane M, Almire F, Kean J, Donald CL, McDonald A, Wee B, et al. The Aedes aegypti Domino Ortholog p400 Regulates Antiviral Exogenous Small Interfering RNA Pathway Activity and ago-2 Expression. mSphere. 2020;5(2). doi: 10.1128/mSphere.00081-20 32269152 PMC7142294

[33] Blair CD, Olson KE. The role of RNA interference (RNAi) in arbovirus-vector interactions. Viruses. 2015;7(2):820–43. doi: 10.3390/v7020820 25690800 PMC4353918

[34] Dostert C, Jouanguy E, Irving P, Troxler L, Galiana-Arnoux D, Hetru C, et al. The Jak-STAT signaling pathway is required but not sufficient for the antiviral response of drosophila. Nat Immunol. 2005;6(9):946–53. doi: 10.1038/ni1237 16086017

[35] Zou Z, Shin SW, Alvarez KS, Bian G, Kokoza V, Raikhel AS. Mosquito RUNX4 in the immune regulation of PPO gene expression and its effect on avian malaria parasite infection. Proc Natl Acad Sci U S A. 2008;105(47):18454–9. doi: 10.1073/pnas.0804658105 19011100 PMC2587535

[36] Marinotti J, James AA. An α- glucosidase in the salivary glands of the vector mosquito, Aedes aegypti. Insect Biochem. 1990;20(6):619–23.

[37] Burkett DA, Carlson DA, Kline DL. Analysis of composition of sugar meals of wild mosquitoes by gas chromatography. J Am Mosq Control Assoc. 1998;14(4):373–9. 10084129

[38] Harris EV, de Roode JC, Gerardo NM. Diet-microbiome-disease: Investigating diet’s influence on infectious disease resistance through alteration of the gut microbiome. PLoS Pathog. 2019;15(10):e1007891. doi: 10.1371/journal.ppat.1007891 31671152 PMC6822718

[39] Satokari R. High Intake of Sugar and the Balance between Pro- and Anti-Inflammatory Gut Bacteria. Nutrients. 2020;12(5). 10.3390/nu12051348 PMC7284805 32397233

[40] Singh RK, Chang HW, Yan D, Lee KM, Ucmak D, Wong K, et al. Influence of diet on the gut microbiome and implications for human health. J Transl Med. 2017;15(1):73. doi: 10.1186/s12967-017-1175-y 28388917 PMC5385025

[41] Jupatanakul N, Sim S, Dimopoulos G. The Insect Microbiome Modulates Vector Competence for Arboviruses. Viruses. 2014;6(11):4294–313. doi: 10.3390/v6114294 25393895 PMC4246223

[42] Rodgers FH, Gendrin M, Christophides GK. Chapter 6—The Mosquito Immune System and Its Interactions With the Microbiota: Implications for Disease Transmission. In: Wikel SK, Aksoy S, Dimopoulos G, editors. Arthropod Vector: Controller of Disease Transmission, Volume 1: Academic Press; 2017. p. 101–22.

[43] Ramirez JL, Souza-Neto J, Torres Cosme R, Rovira J, Ortiz A, Pascale JM, et al. Reciprocal tripartite interactions between the Aedes aegypti midgut microbiota, innate immune system and dengue virus influences vector competence. PLoS Negl Trop Dis. 2012;6(3):e1561. doi: 10.1371/journal.pntd.0001561 22413032 PMC3295821

[44] Franz AW, Kantor AM, Passarelli AL, Clem RJ. Tissue Barriers to Arbovirus Infection in Mosquitoes. Viruses. 2015;7(7):3741–67. doi: 10.3390/v7072795 26184281 PMC4517124

[45] Sanders HR, Evans AM, Ross LS, Gill SS. Blood meal induces global changes in midgut gene expression in the disease vector, Aedes aegypti. Insect biochemistry and molecular biology. 2003;33(11):1105–22. doi: 10.1016/s0965-1748(03)00124-3 14563362

[46] Winokur OC, Main BJ, Nicholson J, Barker CM. Impact of temperature on the extrinsic incubation period of Zika virus in Aedes aegypti. PLoS Negl Trop Dis. 2020;14(3):e0008047. doi: 10.1371/journal.pntd.0008047 32187187 PMC7105136

[47] Ryckebusch F, Berthet M, Misse D, Choumet V. Infection of a French Population of Aedes albopictus and of Aedes aegypti (Paea Strain) with Zika Virus Reveals Low Transmission Rates to These Vectors’ Saliva. Int J Mol Sci. 2017;18(11). doi: 10.3390/ijms18112384 29125545 PMC5713353

[48] Dubrulle M, Mousson L, Moutailler S, Vazeille M, Failloux AB. Chikungunya virus and Aedes mosquitoes: saliva is infectious as soon as two days after oral infection. PloS one. 2009;4(6):e5895. doi: 10.1371/journal.pone.0005895 19521520 PMC2690823

[49] Nobs SP, Zmora N, Elinav E. Nutrition Regulates Innate Immunity in Health and Disease. Annu Rev Nutr. 2020;40:189–219. doi: 10.1146/annurev-nutr-120919-094440 32520640

[50] Weger-Lucarelli J, Carrau L, Levi LI, Rezelj V, Vallet T, Blanc H, et al. Host nutritional status affects alphavirus virulence, transmission, and evolution. PLoS Pathog. 2019;15(11):e1008089. doi: 10.1371/journal.ppat.1008089 31710653 PMC6872174

[51] Xu J, Hopkins K, Sabin L, Yasunaga A, Subramanian H, Lamborn I, et al. ERK signaling couples nutrient status to antiviral defense in the insect gut. Proc Natl Acad Sci U S A. 2013;110(37):15025–30. doi: 10.1073/pnas.1303193110 23980175 PMC3773808

[52] Koella JC, Sorense FL. Effect of adult nutrition on the melanization immune response of the malaria vector Anopheles stephensi. Medical and veterinary entomology. 2002;16(3):316–20. doi: 10.1046/j.1365-2915.2002.00381.x 12243233

[53] Ferguson LV, Beckett NH, French M-C, Campbell MJ, Smith TG, Adamo SA. Sugar intake interacts with temperature to influence reproduction and Immunity in adult Culex pipiens mosquitoes. Can J Zool. 2019;97:424–8.

[54] Ponton F, Morimoto J, Robinson K, Kumar SS, Cotter SC, Wilson K, et al. Macronutrients modulate survival to infection and immunity in Drosophila. J Anim Ecol. 2020;89(2):460–70. doi: 10.1111/1365-2656.13126 31658371 PMC7027473

[55] Galenza A, Hutchinson J, Campbell SD, Hazes B, Foley E. Glucose modulates Drosophila longevity and immunity independent of the microbiota. Biol Open. 2016;5(2):165–73. doi: 10.1242/bio.015016 26794610 PMC4823985

[56] Kay AD, Bruning AJ, van Alst A, Abrahamson TT, Hughes WO, Kaspari M. A carbohydrate-rich diet increases social immunity in ants. Proc Biol Sci. 2014;281(1778):20132374. doi: 10.1098/rspb.2013.2374 24430844 PMC3906932

[57] Wang A, Huen SC, Luan HH, Yu S, Zhang C, Gallezot JD, et al. Opposing Effects of Fasting Metabolism on Tissue Tolerance in Bacterial and Viral Inflammation. Cell. 2016;166(6):1512–25.e12. doi: 10.1016/j.cell.2016.07.026 27610573 PMC5555589

[58] Sim S, Jupatanakul N, Ramirez JL, Kang S, Romero-Vivas CM, Mohammed H, et al. Transcriptomic profiling of diverse Aedes aegypti strains reveals increased basal-level immune activation in dengue virus-refractory populations and identifies novel virus-vector molecular interactions. PLoS Negl Trop Dis. 2013;7(7):e2295. doi: 10.1371/journal.pntd.0002295 23861987 PMC3701703

[59] Baldini F, Gabrieli P, South A, Valim C, Mancini F, Catteruccia F. The interaction between a sexually transferred steroid hormone and a female protein regulates oogenesis in the malaria mosquito Anopheles gambiae. PLoS biology. 2013;11(10):e1001695. doi: 10.1371/journal.pbio.1001695 24204210 PMC3812110

[60] Luckhart S, Riehle MA. The insulin signaling cascade from nematodes to mammals: insights into innate immunity of Anopheles mosquitoes to malaria parasite infection. Developmental and comparative immunology. 2007;31(7):647–56. doi: 10.1016/j.dci.2006.10.005 17161866 PMC2233911

[61] Corby-Harris V, Drexler A, Watkins de Jong L, Antonova Y, Pakpour N, Ziegler R, et al. Activation of Akt signaling reduces the prevalence and intensity of malaria parasite infection and lifespan in Anopheles stephensi mosquitoes. PLoS Pathog. 2010;6(7):e1001003. doi: 10.1371/journal.ppat.1001003 20664791 PMC2904800

[62] Pakpour N, Corby-Harris V, Green GP, Smithers HM, Cheung KW, Riehle MA, et al. Ingested human insulin inhibits the mosquito NF-kappaB-dependent immune response to Plasmodium falciparum. Infect Immun. 2012;80(6):2141–9. doi: 10.1128/IAI.00024-12 22473605 PMC3370580

[63] Surachetpong W, Singh N, Cheung KW, Luckhart S. MAPK ERK Signaling Regulates the TGF-β1-Dependent Mosquito Response to Plasmodium falciparum. PLOS Pathogens. 2009;5(4):e1000366. doi: 10.1371/journal.ppat.1000366 19343212 PMC2658807

[64] Kang MA, Mott TM, Tapley EC, Lewis EE, Luckhart S. Insulin regulates aging and oxidative stress in Anopheles stephensi. J Exp Biol. 2008;211(Pt 5):741–8. doi: 10.1242/jeb.012955 18281336 PMC2592302

[65] Ganeshan K, Chawla A. Metabolic regulation of immune responses. Annu Rev Immunol. 2014;32:609–34. doi: 10.1146/annurev-immunol-032713-120236 24655299 PMC5800786

[66] Apte-Deshpande A, Paingankar M, Gokhale MD, Deobagkar DN. Serratia odorifera a midgut inhabitant of Aedes aegypti mosquito enhances its susceptibility to dengue-2 virus. PloS one. 2012;7(7):e40401. doi: 10.1371/journal.pone.0040401 22848375 PMC3407224

[67] Wu P, Sun P, Nie K, Zhu Y, Shi M, Xiao C, et al. A Gut Commensal Bacterium Promotes Mosquito Permissiveness to Arboviruses. Cell host & microbe. 2019;25(1):101–12 e5. doi: 10.1016/j.chom.2018.11.004 30595552

[68] Wong AC, Dobson AJ, Douglas AE. Gut microbiota dictates the metabolic response of Drosophila to diet. J Exp Biol. 2014;217(Pt 11):1894–901. doi: 10.1242/jeb.101725 24577449 PMC4037322

[69] Consuegra J, Grenier T, Baa-Puyoulet P, Rahioui I, Akherraz H, Gervais H, et al. Drosophila-associated bacteria differentially shape the nutritional requirements of their host during juvenile growth. PLoS biology. 2020;18(3):e3000681. doi: 10.1371/journal.pbio.3000681 32196485 PMC7112240

[70] Huang JH, Douglas AE. Consumption of dietary sugar by gut bacteria determines Drosophila lipid content. Biol Lett. 2015;11(9):20150469. doi: 10.1098/rsbl.2015.0469 26382071 PMC4614424

[71] Storelli G, Defaye A, Erkosar B, Hols P, Royet J, Leulier F. Lactobacillus plantarum promotes Drosophila systemic growth by modulating hormonal signals through TOR-dependent nutrient sensing. Cell Metab. 2011;14(3):403–14. doi: 10.1016/j.cmet.2011.07.012 21907145

[72] Shin SC, Kim SH, You H, Kim B, Kim AC, Lee KA, et al. Drosophila microbiome modulates host developmental and metabolic homeostasis via insulin signaling. Science. 2011;334(6056):670–4. doi: 10.1126/science.1212782 22053049

[73] Chabanol E, Behrends V, Prevot G, Christophides GK, Gendrin M. Antibiotic Treatment in Anopheles coluzzii Affects Carbon and Nitrogen Metabolism. Pathogens. 2020;9(9). doi: 10.3390/pathogens9090679 32825534 PMC7558193

[74] Smith SM, Gadawski RM. Nectar feeding by the early-spring mosquito Aedes provocans. Medical and veterinary entomology. 1994;8(3):201–13. doi: 10.1111/j.1365-2915.1994.tb00499.x 7949310

[75] Andersson IH. Nectar feeding activity of Aedes mosquitoes, with special reference to Aedes communis females. J Am Mosq Control Assoc. 1990;6(3):482–9. 2230777

[76] Martinez-Ibarra JA, Rodriguez MH, Arredondo-Jimenez JI, Yuval B. Influence of plant abundance on nectar feeding by Aedes aegypti (Diptera: Culicidae) in southern Mexico. Journal of medical entomology. 1997;34(6):589–93. doi: 10.1093/jmedent/34.6.589 9439110

[77] Edman JD, Strickman D, Kittayapong P, Scott TW. Female Aedes aegypti (Diptera: Culicidae) in Thailand rarely feed on sugar. Journal of medical entomology. 1992;29(6):1035–8. doi: 10.1093/jmedent/29.6.1035 1460619

[78] Scott TW, Chow E, Strickman D, Kittayapong P, Wirtz RA, Lorenz LH, et al. Blood-feeding patterns of Aedes aegypti (Diptera: Culicidae) collected in a rural Thai village. Journal of medical entomology. 1993;30(5):922–7. doi: 10.1093/jmedent/30.5.922 8254642

[79] Ignell R, Okawa S, Englund J-E, Hill SR. Assessment of diet choice by the yellow fever mosquito Aedes aegypti. Physiological Entomology. 2010;35(274–286).

[80] Scott TW, Amerasinghe PH, Morrison AC, Lorenz LH, Clark GG, Strickman D, et al. Longitudinal studies of Aedes aegypti (Diptera: Culicidae) in Thailand and Puerto Rico: blood feeding frequency. Journal of medical entomology. 2000;37(1):89–101. doi: 10.1603/0022-2585-37.1.89 15218911

[81] Reyes-Villanueva F. Egg development may require multiple bloodmeals among small Aedes aegypti (Diptera: culicidae) field collected in northeastern Mexico. Florida Entomogist. 2004;87(4).

[82] Farjana T, Tuno N. Multiple blood feeding and host-seeking behavior in Aedes aegypti and Aedes albopictus (Diptera: Culicidae). Journal of medical entomology. 2013;50(4):838–46. doi: 10.1603/me12146 23926783

[83] McFarlane M, Laureti M, Levee T, Terry S, Kohl A, Pondeville E. Improved transient silencing of gene expression in the mosquito female Aedes aegypti. Insect molecular biology. 2021;30(3):355–65. doi: 10.1111/imb.12700 33715239

[84] Hale BG, Knebel A, Botting CH, Galloway CS, Precious BL, Jackson D, et al. CDK/ERK-mediated phosphorylation of the human influenza A virus NS1 protein at threonine-215. Virology. 2009;383(1):6–11. doi: 10.1016/j.virol.2008.10.002 19007960

[85] Hilton L, Moganeradj K, Zhang G, Chen YH, Randall RE, McCauley JW, et al. The NPro product of bovine viral diarrhea virus inhibits DNA binding by interferon regulatory factor 3 and targets it for proteasomal degradation. J Virol. 2006;80(23):11723–32. doi: 10.1128/JVI.01145-06 16971436 PMC1642611

[86] Ulper L, Sarand I, Rausalu K, Merits A. Construction, properties, and potential application of infectious plasmids containing Semliki Forest virus full-length cDNA with an inserted intron. J Virol Methods. 2008;148(1–2):265–70. doi: 10.1016/j.jviromet.2007.10.007 18054090 PMC7172237

[87] Mutso M, Saul S, Rausalu K, Susova O, Zusinaite E, Mahalingam S, et al. Reverse genetic system, genetically stable reporter viruses and packaged subgenomic replicon based on a Brazilian Zika virus isolate. The Journal of general virology. 2017;98(11):2712–24. doi: 10.1099/jgv.0.000938 29022864

[88] Taylor SC, Nadeau K, Abbasi M, Lachance C, Nguyen M, Fenrich J. The Ultimate qPCR Experiment: Producing Publication Quality, Reproducible Data the First Time. Trends Biotechnol. 2019;37(7):761–74. doi: 10.1016/j.tibtech.2018.12.002 30654913

