# Supplementary material for: Sugar feeding protects against arboviral infection by enhancing gut immunity in the mosquito vector Aedes aegypti
Source: PLoS Pathog. 2021 Sep 2;17(9):e1009870. doi: 10.1371/journal.ppat.1009870 (PMC8412342; doi:10.1371/journal.ppat.1009870)
Supplement: S1 Fig — Pictures of digestive tracts dissected from females fed with a blue-stained 10% sucrose solution. (A) Picture of a representative gut minutes after sugar feeding showing sugar stored in the crop and relocated to midgut. (B) Picture of a representative gut one day after sugar feeding, showing sugar in the crop (although less than just after feeding) and little to no sugar solution in the midgut. (DOCX) [file ppat.1009870.s001.docx]

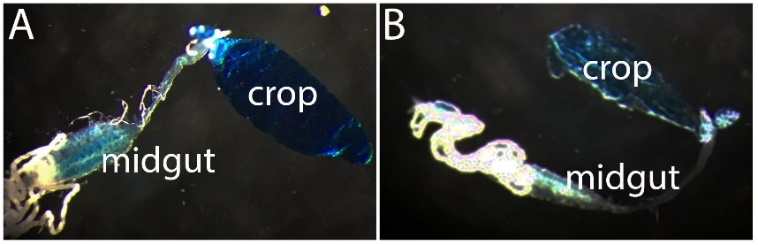


**S1 Fig. Sucrose solution is stored in the crop and intermittently relocated to the midgut.** Pictures of digestive tracts dissected from females fed with a blue-stained 10% sucrose solution. (A) Picture of a representative gut minutes after sugar feeding showing sugar stored in the crop and relocated to midgut. (B) Picture of a representative gut one day after sugar feeding, showing sugar in the crop (although less than just after feeding) and little to no sugar solution in the midgut.
