# Supplementary material for: Sugar feeding protects against arboviral infection by enhancing gut immunity in the mosquito vector Aedes aegypti
Source: PLoS Pathog. 2021 Sep 2;17(9):e1009870. doi: 10.1371/journal.ppat.1009870 (PMC8412342; doi:10.1371/journal.ppat.1009870)
Supplement: S2 Fig — Females, previously treated or not with antibiotics, did not have access to sucrose for 48 h to empty their crops and were then either not fed or fed with 10% sucrose. Digestive tracts were dissected (A to D) 16 h or (E) 18 h post sugar feeding time (E). (A to D) LB agar plates after plating homogenised and diluted (1/100) digestive tracts from (A) non fed females and (B) sucrose fed females not treated with antibiotics and digestive tracts from (C) non-fed females and (D) sucrose fed females treated with antibiotics. (E) 16S relative gene levels in digestive tracts from the four populations, relative to S7 ribosomal protein gene, were analysed by qPCR. RQ for each sample was obtained as described (88), normalised to the S7 ribosomal gene and as relative values to that of the respective control group (not treated with antibiotics, RQ geomean set to 1). Box plots display the RQ minimum, first quartile, median, third quartile, and maximum. Log2-transformed RQ values were analysed by ANOVA on matched values followed by a Holm-Sidak’s multiple comparison test. Bacteria in the digestive tracts of antibiotic-treated females were significantly depleted. Samples were similarly depleted of bacteria (No sucrose vs Sucrose: ns). N = 3 pools (from 3 independent experiments) of 5 digestive tracts per condition. (DOCX) [file ppat.1009870.s002.docx]

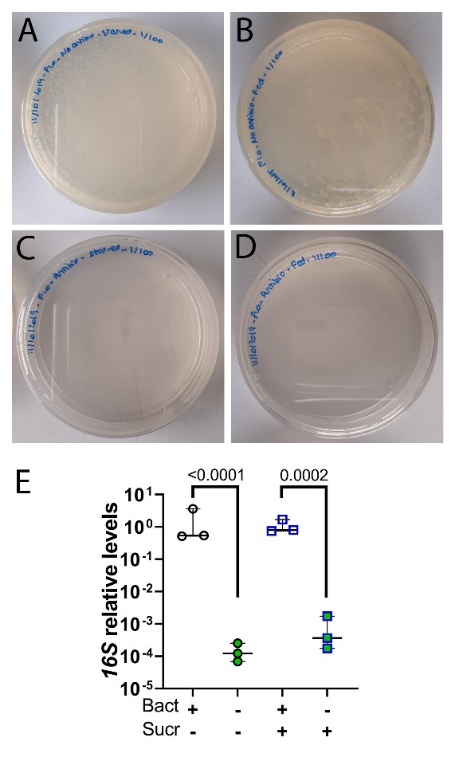


**S2 Fig. Validation of antibiotic treatment efficacy.** Females, previously treated or not with antibiotics, did not have access to sucrose for 48 h to empty their crops and were then either not fed or fed with 10% sucrose. Digestive tracts were dissected (A to D) 16 h or (E) 18 h post sugar feeding time (E). (A to D) LB agar plates after plating homogenised and diluted (1/100) digestive tracts from (A) non fed females and (B) sucrose fed females not treated with antibiotics and digestive tracts from (C) non-fed females and (D) sucrose fed females treated with antibiotics. (E) *16S* relative gene levels in digestive tracts from the four populations, relative to *S7 ribosomal protein* gene, were analysed by qPCR. RQ for each sample was obtained as described (1), normalised to the *S7* ribosomal gene and as relative values to that of the respective control group (not treated with antibiotics, RQ geomean set to 1). Box plots display the RQ minimum, first quartile, median, third quartile, and maximum. Log2-transformed RQ values were analysed by ANOVA on matched values followed by a Holm-Sidak’s multiple comparison test. Bacteria in the digestive tracts of antibiotic-treated females were significantly depleted. Samples were similarly depleted of bacteria (No sucrose vs Sucrose: ns). N = 3 pools (from 3 independent experiments) of 5 digestive tracts per condition.

1. Taylor SC, Nadeau K, Abbasi M, Lachance C, Nguyen M, Fenrich J. The Ultimate qPCR Experiment: Producing Publication Quality, Reproducible Data the First Time. Trends Biotechnol. 2019;37(7):761-74.
