# Supplementary material for: Sugar feeding protects against arboviral infection by enhancing gut immunity in the mosquito vector Aedes aegypti
Source: PLoS Pathog. 2021 Sep 2;17(9):e1009870. doi: 10.1371/journal.ppat.1009870 (PMC8412342; doi:10.1371/journal.ppat.1009870)
Supplement: S3 Fig — Females previously treated with antibiotics did not have access to sucrose for 48 h and were either not fed (NSF), fed with 10% sucrose (SF), or blood fed (BF). Digestive tracts were dissected 16 h post feeding time. RNA transcript levels of p400, piwi4 and ppo8. Box plots display the minimum, first quartile, median, third quartile, and maximum relative expression levels. N = 5 pools of 5 digestive tracts per condition. Statistical significance was assessed with an analysis of variance followed by a Fisher’s multiple comparison test. ns, p value > 0.05; *, p value < 0.05; ****, p value <0.0001. (DOCX) [file ppat.1009870.s003.docx]

**
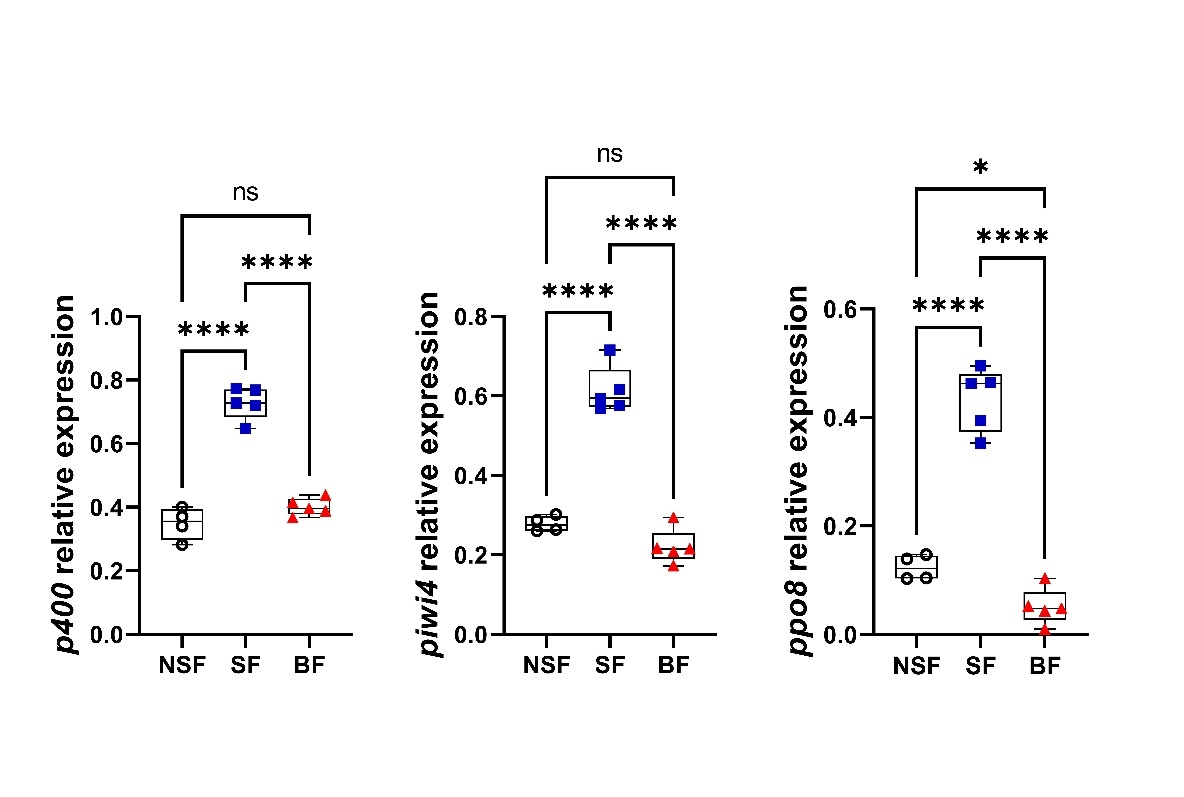
**

**S3 Fig.** **Immunity is not upregulated in aseptic blood fed females.** Females previously treated with antibiotics did not have access to sucrose for 48 h and were either not fed (NSF), fed with 10% sucrose (SF), or blood fed (BF). Digestive tracts were dissected 16 h post feeding time. RNA transcript levels of *p400*, *piwi4* and *ppo8*. Box plots display the minimum, first quartile, median, third quartile, and maximum relative expression levels. N = 5 pools of 5 digestive tracts per condition. Statistical significance was assessed with an analysis of variance followed by a Fisher’s multiple comparison test. ns, p value > 0.05; *, p value < 0.05; ****, p value <0.0001.
