# Supplementary material for: Sugar feeding protects against arboviral infection by enhancing gut immunity in the mosquito vector Aedes aegypti
Source: PLoS Pathog. 2021 Sep 2;17(9):e1009870. doi: 10.1371/journal.ppat.1009870 (PMC8412342; doi:10.1371/journal.ppat.1009870)
Supplement: S4 Fig — Treatments: Sugar and Antibiotic (Bacteria) treatments. Significant treatment effects and interaction between treatments (p values < 0.05) are highlighted in blue. (DOCX) [file ppat.1009870.s004.docx]

**
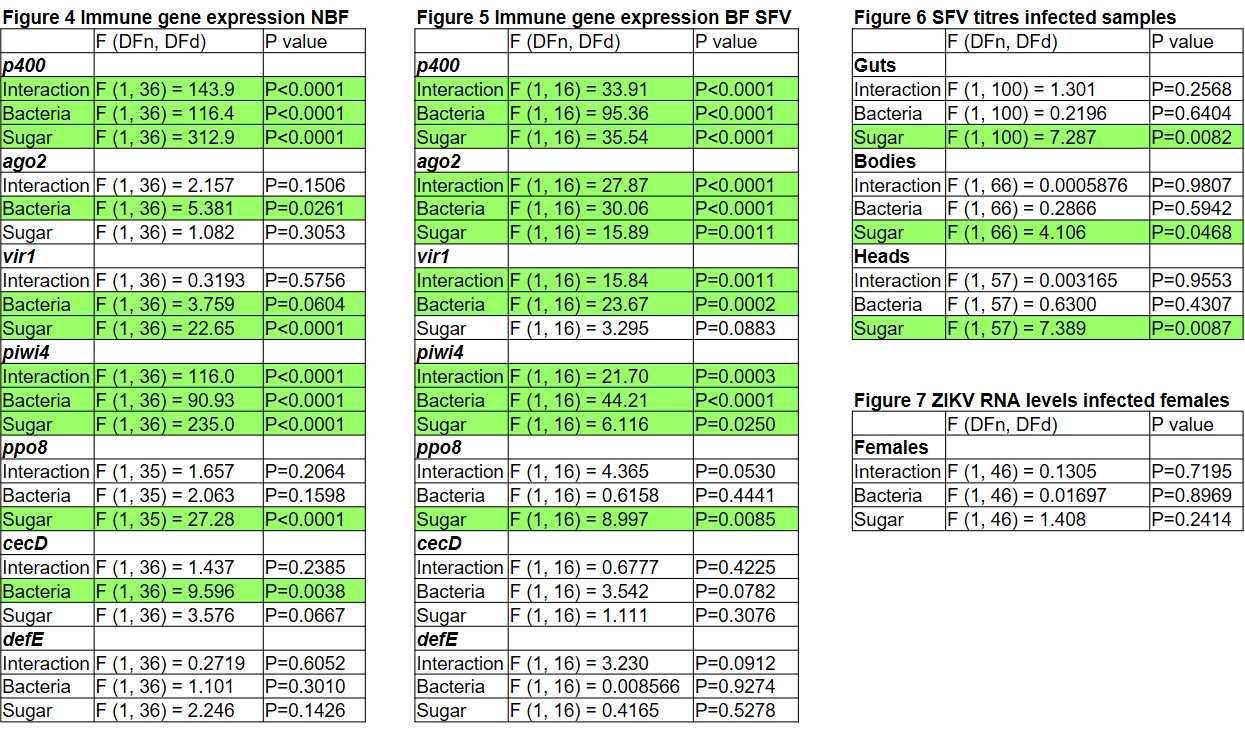
**

**S4 Fig. Two-way ANOVA statistical significance of treatments and interaction between treatments.** Treatments: Sugar and Antibiotic (Bacteria) treatments. Significant treatment effects and interaction between treatments (p values < 0.05) are highlighted in blue.
