# Supplementary material for: Sugar feeding protects against arboviral infection by enhancing gut immunity in the mosquito vector Aedes aegypti
Source: PLoS Pathog. 2021 Sep 2;17(9):e1009870. doi: 10.1371/journal.ppat.1009870 (PMC8412342; doi:10.1371/journal.ppat.1009870)
Supplement: S5 Fig — SFV infection, dissemination and transmission potential prevalence (in percentage and numbers in brackets). The p values indicate statistical significance of the treatments effect on prevalence assessed with a Chi-square test (compared to No bacteria—No sucrose group). ns, p value > 0.05. Data corresponding to graph presented on Fig 6E. (DOCX) [file ppat.1009870.s005.docx]

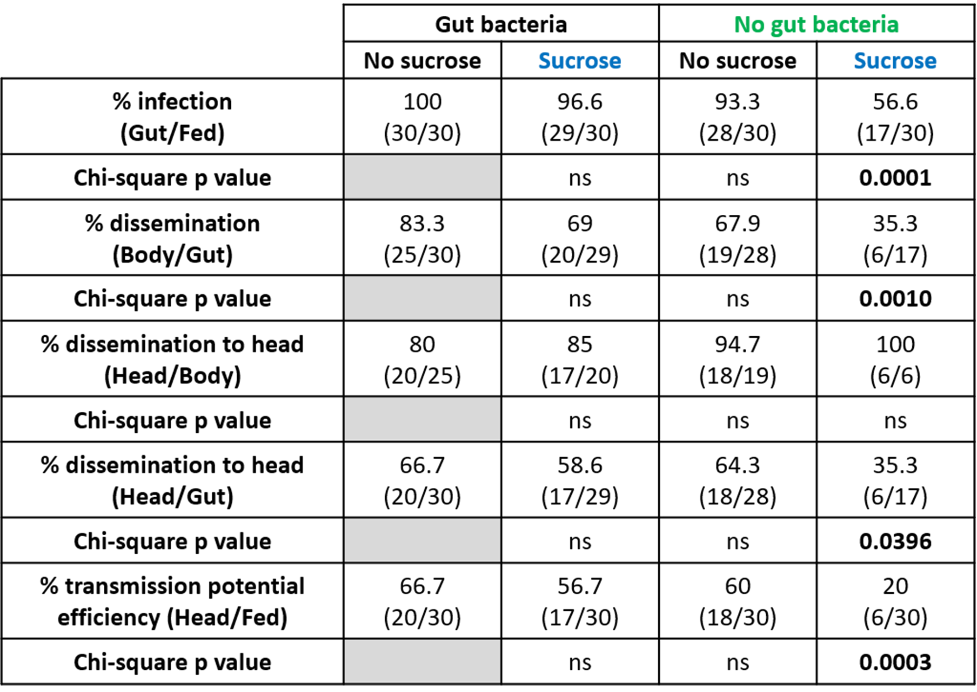


**S5 Fig. Sugar feeding protects the female mosquito *Ae. aegypti* against SFV infection.** SFV infection, dissemination and transmission potential prevalence (in percentage and numbers in brackets). The p values indicate statistical significance of the treatments effect on prevalence assessed with a Chi-square test (compared to No bacteria - No sucrose group). ns, p value > 0.05. Data corresponding to graph presented on Fig 6E.
