# Supplementary material for: Sugar feeding protects against arboviral infection by enhancing gut immunity in the mosquito vector Aedes aegypti
Source: PLoS Pathog. 2021 Sep 2;17(9):e1009870. doi: 10.1371/journal.ppat.1009870 (PMC8412342; doi:10.1371/journal.ppat.1009870)
Supplement: S6 Fig — ZIKV infection prevalence (in percentage and numbers in brackets). The p values indicate statistical significance of the treatment effect on prevalence assessed with a Chi-square test (compared to No bacteria- No sucrose group). Data corresponding to graph presented on Fig 7B. (DOCX) [file ppat.1009870.s006.docx]

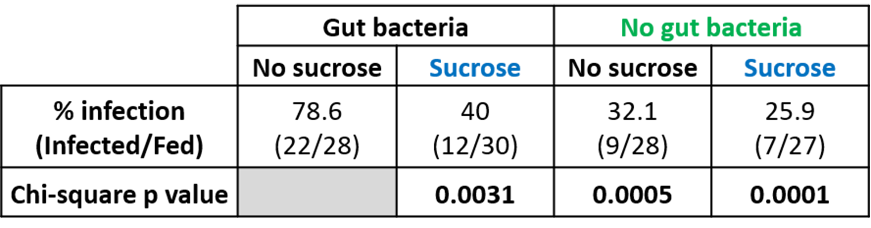


**S6 Fig. Sugar feeding protects the female mosquito *Ae. aegypti* against ZIKV infection.** ZIKV infection prevalence (in percentage and numbers in brackets). The p values indicate statistical significance of the treatment effect on prevalence assessed with a Chi-square test (compared to No bacteria- No sucrose group). Data corresponding to graph presented on Fig 7B.
