# Supplementary material for: Sugar feeding protects against arboviral infection by enhancing gut immunity in the mosquito vector Aedes aegypti
Source: PLoS Pathog. 2021 Sep 2;17(9):e1009870. doi: 10.1371/journal.ppat.1009870 (PMC8412342; doi:10.1371/journal.ppat.1009870)
Supplement: S7 Fig — Females treated with antibiotics, were injected with dsRNA targeting either luciferase (dsLuc, control) or targeting runx4, piwi4, dcr2 and myd88 (dsImm). Two days later, females were starved before being fed with 10% sucrose. Digestive tracts were dissected 16 h post sugar feeding time. RNA transcript levels of runx4, ppo8, piwi4, dcr2 and myd88. Box plots display the minimum, first quartile, median, third quartile, and maximum relative expression levels. N = 5 pools of 5 digestive tracts per condition. Statistical significance of the treatment effect was assessed with an unpaired t test two-tailed. ns, p value > 0.05; **, p value < 0.01; ****, p value <0.0001. (DOCX) [file ppat.1009870.s007.docx]

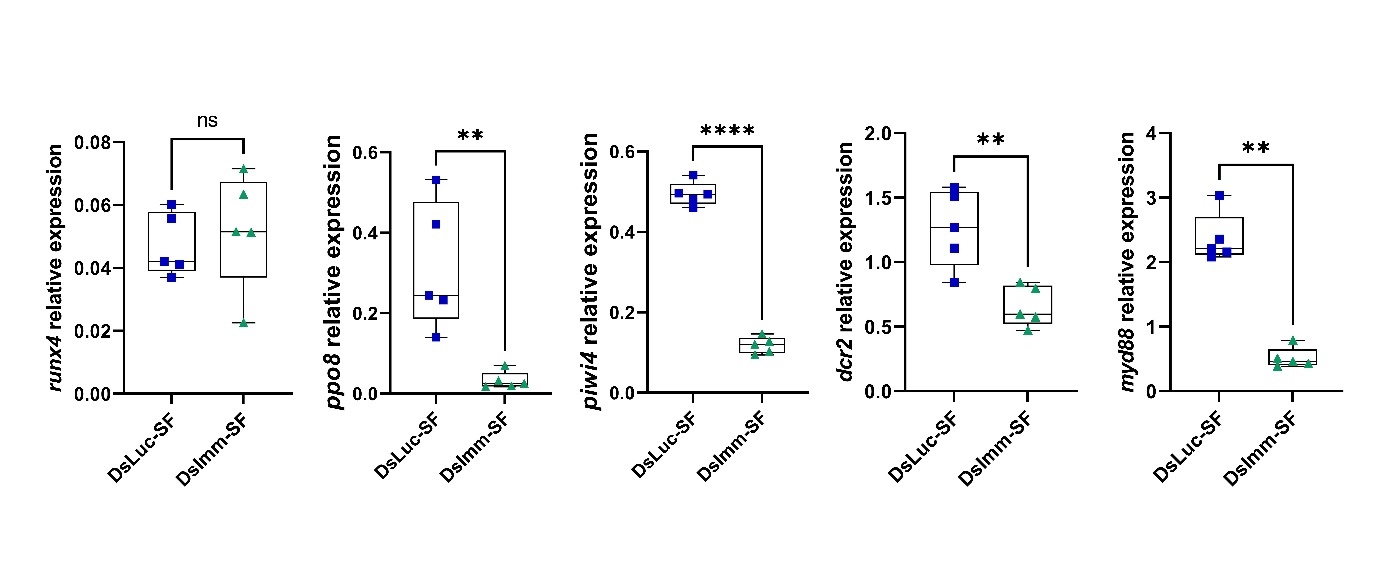


**S7 Fig. Knock down efficiency of *ppo8*, *piwi4*, *dcr2* and *myd88*.** Females treated with antibiotics, were injected with dsRNA targeting either luciferase (dsLuc, control) or targeting *runx4*, *piwi4*, *dcr2* and *myd88* (dsImm). Two days later, females were starved before being fed with 10% sucrose. Digestive tracts were dissected 16 h post sugar feeding time. RNA transcript levels of *runx4*, *ppo8*, *piwi4*, *dcr2* and *myd88*. Box plots display the minimum, first quartile, median, third quartile, and maximum relative expression levels. N = 5 pools of 5 digestive tracts per condition. Statistical significance of the treatment effect was assessed with an unpaired t test two-tailed. ns, p value > 0.05; **, p value < 0.01; ****, p value <0.0001.
