# Supplementary material for: Sugar feeding protects against arboviral infection by enhancing gut immunity in the mosquito vector Aedes aegypti
Source: PLoS Pathog. 2021 Sep 2;17(9):e1009870. doi: 10.1371/journal.ppat.1009870 (PMC8412342; doi:10.1371/journal.ppat.1009870)
Supplement: S8 Fig — SFV infection prevalence (in percentage and numbers in brackets). The p values indicate statistical significance of the treatment effect on prevalence assessed with a Chi-square test (compared to DsLuc-NSF group). Data corresponding to graph presented on Fig 8B. (DOCX) [file ppat.1009870.s008.docx]

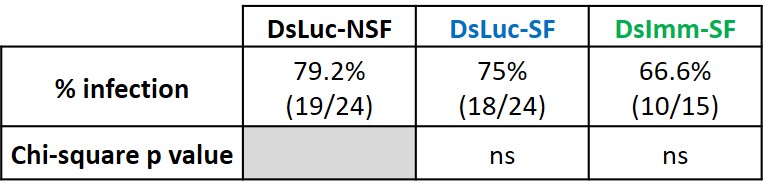


**S8 Fig. Sugar-mediated protection against arboviral infection is mediated by sugar-enhanced immunity.** SFV infection prevalence (in percentage and numbers in brackets). The p values indicate statistical significance of the treatment effect on prevalence assessed with a Chi-square test (compared to dsLuc-NSF group). Data corresponding to graph presented on Fig 8B.
