# Supplementary material for: Sugar feeding protects against arboviral infection by enhancing gut immunity in the mosquito vector Aedes aegypti
Source: PLoS Pathog. 2021 Sep 2;17(9):e1009870. doi: 10.1371/journal.ppat.1009870 (PMC8412342; doi:10.1371/journal.ppat.1009870)
Supplement: S1 Table — (DOCX) [file ppat.1009870.s009.docx]

**S1 Table. List of primers used in this study.**

| **Gene** | **Primers** | **Use** | **Reference** |
| --- | --- | --- | --- |
| ***S7*** | **qPCR S7 F** CCAGGCTATCCTGGAGTTG  **qPCR S7 R** GACGTGCTTGCCGGAGAAC | qPCR | (1) |
| ***p400*** | **p400qFOR** GGAACCAGTCCAGCCATGAA  **p400qREV** CGATCGCTCCTGCATTTGTG | qPCR | (2) |
| ***ago2*** | **qPCR ago2 F** GGCTGCTCACCCAATGTATCAAGA  **qPCR ago2 R** AACCGTTCGTTTTGGCGTTGAT | qPCR | (1) |
| ***piwi4*** | **piwi4qFOR2** TACGACATCTTGCGCGATTG  **piwi4qREV2** CCAAGTACGGCACGTTTGAA | qPCR | This study |
| ***ppo8*** | **ppo8qFOR** GCTTTGCTATGTCCGCCAAT  **ppo8qREV** CGCATTCGGAGACAATGATG | qPCR | This study |
| ***vir1*** | **vir1qFOR** CCATCGGATGACACGGAGTA  **vir1qREV** GTGTCCACAATGCCATCGAA | qPCR | This study |
| ***cecD*** | **cecDqFOR** CGCTTTGGTCCTGCTAGGT  **cecDqREV** AAGCCTTGAATACTCGCTTGC | qPCR | This study |
| ***defE*** | **defEqFOR** AACGTCGAAAGCGCATCTCA  **defEqREV** CGGTAGCGCCAGCTTATGG | qPCR | (3) |
| ***ZIKV***  ***(NS3)*** | **qZIKVNS3F1** ATCTGTATGGAGGTGGGTGC  **qZIKVNS3R1** CTCTCCCTCAATGGCTGCTA | qPCR | This study |
| ***16S*** | **16SF** TCCTACGGGAGGCAGCAGT  **16SR** GGACTACCAGGGTATCTAATCCTGTT | qPCR | (4) |
| ***dsLuc*** | **dsLuc-T7-FOR**  taatacgactcactatagggATGGAAGACGCCA AAAACATAAA  **dsLuc-T7-REV** taatacgactcactatagggAGAGAGTTTTCACTGCATACG | dsRNA synthesis | This study |
| ***dsMyd88*** | **dsMYD88-T7-FOR** taatacgactcactatagggGGCGATTGGTGGTTGTTATT  **dsMYD88-T7-REV** taatacgactcactatagggTTGAGCGCATTGCTAACATC | dsRNA synthesis | (5) |
| ***dsDcr2*** | **dsDcr2-T7-FOR**  taatacgactcactatagggTCAGCTTGAGCTGCATGGC  **dsDcr2-T7-REV**  taatacgactcactatagggTCTGTAGTTCTTGAGGAATGC | dsRNA synthesis | This study |
| ***dsPiwi4*** | **dsPiwi4-T7-FOR** taatacgactcactatagggGTTCGACGTTTGCCATGAT  **dsPiwi4-T7-REV** taatacgactcactatagggAGATGATACGTTTGGGCAGC | dsRNA synthesis | This study |
| ***dsRUNX4*** | **dsRUNX4-T7-FOR** taatacgactcactatagggCCACCGATCAGTAACAACGA  **dsRUNX4-T7-REV** taatacgactcactatagggGTCACCTTGATGGCTTTGGT | dsRNA synthesis | (6) |
| ***myd88KDeff*** | **MYD88KDeffQFOR** AACTGCTGTCGGCATTGCTA  **MYD88KDeffQREV** GAAACAACCCTCGCCAATCAC | qPCR | This study |
| ***dcr2*** | **dcr2 QFOR** ACCCACGTGTAATCGGTCTT  **dcr2QREV** CGGTAGCAATTGTCGCGTTA | qPCR | This study |
| ***RUNX4KDeff*** | **RUNX4KDeffQFOR** GTGGGCCAACGGATATGGAA  **RUNX4KDeffQREV** CGGGTGCAGTGGAAGGATAC | qPCR | This study |

**References**

1. McFarlane M, Arias-Goeta C, Martin E, O'Hara Z, Lulla A, Mousson L, et al. Characterization of *Aedes aegypti* innate-immune pathways that limit Chikungunya virus replication. PLoS Negl Trop Dis. 2014;8(7):e2994.

2. McFarlane M, Almire F, Kean J, Donald CL, McDonald A, Wee B, et al. The Aedes aegypti Domino Ortholog p400 Regulates Antiviral Exogenous Small Interfering RNA Pathway Activity and ago-2 Expression. mSphere. 2020;5(2).

3. Jupatanakul N, Sim S, Dimopoulos G. Aedes aegypti ML and Niemann-Pick type C family members are agonists of dengue virus infection. Developmental and comparative immunology. 2014;43(1):1-9.

4. Wei G, Lai Y, Wang G, Chen H, Li F, Wang S. Insect pathogenic fungus interacts with the gut microbiota to accelerate mosquito mortality. Proc Natl Acad Sci U S A. 2017;114(23):5994-9.

5. Xi Z, Ramirez JL, Dimopoulos G. The *Aedes aegypti* toll pathway controls dengue virus infection. PLoS Pathog. 2008;4(7):e1000098.

6. Zou Z, Shin SW, Alvarez KS, Bian G, Kokoza V, Raikhel AS. Mosquito RUNX4 in the immune regulation of PPO gene expression and its effect on avian malaria parasite infection. Proc Natl Acad Sci U S A. 2008;105(47):18454-9.
